# RNA-Seq analysis of chikungunya virus infection and identification of granzyme A as a major promoter of arthritic inflammation

**DOI:** 10.1371/journal.ppat.1006155

**Published:** 2017-02-16

**Authors:** Jane A. C. Wilson, Natalie A. Prow, Wayne A. Schroder, Jonathan J. Ellis, Helen E. Cumming, Linden J. Gearing, Yee Suan Poo, Adam Taylor, Paul J. Hertzog, Francesca Di Giallonardo, Linda Hueston, Roger Le Grand, Bing Tang, Thuy T. Le, Joy Gardner, Suresh Mahalingam, Pierre Roques, Phillip I. Bird, Andreas Suhrbier

**Affiliations:** 1 QIMR Berghofer Medical Research Institute, Brisbane, Queensland, Australia; 2 Australian Infectious Disease Research Centre, The University of Queensland, Brisbane, Queensland, Australia; 3 Hudson Institute of Medical Research, Clayton, Victoria, Australia; 4 Institute for Glycomics, Griffith University, Gold Coast, Queensland, Australia; 5 Charles Perkins Centre, The University of Sydney, Sydney, NSW, Australia; 6 Centre for Infectious Diseases and Microbiology Laboratory Services, Westmead Hospital, Sydney, NSW, Australia; 7 CEA, Inserm, Université Paris Sud; iMETI; UMR 1184 Immunology of Viral infections and Autoimmune diseases, Fontenay-aux-Roses, France; 8 Biomedicine Discovery Institute, Monash University, Victoria 3800, Australia; University of North Carolina at Chapel Hill, UNITED STATES

## Abstract

Chikungunya virus (CHIKV) is an arthritogenic alphavirus causing epidemics of acute and chronic arthritic disease. Herein we describe a comprehensive RNA-Seq analysis of feet and lymph nodes at peak viraemia (day 2 post infection), acute arthritis (day 7) and chronic disease (day 30) in the CHIKV adult wild-type mouse model. Genes previously shown to be up-regulated in CHIKV patients were also up-regulated in the mouse model. CHIKV sequence information was also obtained with up to ≈8% of the reads mapping to the viral genome; however, no adaptive viral genome changes were apparent. Although day 2, 7 and 30 represent distinct stages of infection and disease, there was a pronounced overlap in up-regulated host genes and pathways. Type I interferon response genes (IRGs) represented up to ≈50% of up-regulated genes, even after loss of type I interferon induction on days 7 and 30. Bioinformatic analyses suggested a number of interferon response factors were primarily responsible for maintaining type I IRG induction. A group of genes prominent in the RNA-Seq analysis and hitherto unexplored in viral arthropathies were granzymes A, B and K. Granzyme A^-/-^ and to a lesser extent granzyme K^-/-^, but not granzyme B^-/-^, mice showed a pronounced reduction in foot swelling and arthritis, with analysis of granzyme A^-/-^ mice showing no reductions in viral loads but reduced NK and T cell infiltrates post CHIKV infection. Treatment with Serpinb6b, a granzyme A inhibitor, also reduced arthritic inflammation in wild-type mice. In non-human primates circulating granzyme A levels were elevated after CHIKV infection, with the increase correlating with viral load. Elevated granzyme A levels were also seen in a small cohort of human CHIKV patients. Taken together these results suggest granzyme A is an important driver of arthritic inflammation and a potential target for therapy.

**Trial Registration:** ClinicalTrials.gov NCT00281294

## Author summary

The largest chikungunya virus (CHIKV) epidemic ever recorded began in 2004 in Africa and spread across Asia reaching Europe and recently the Americas, with millions of cases reported. We undertook a detailed analysis of the mRNA expression profile during acute and chronic arthritis in an adult wild-type mouse model of CHIKV infection and disease. Gene induction profiles showed a high concordance with published human data, providing some validation of the mouse model. The host response was overwhelmingly dominated by type I interferon response genes, even after type I interferon induction was lost. The analysis also provided information on CHIKV RNA, with no adaptive viral genome changes identified. An important goal of the analysis was to identify new players in arthritic inflammation. Granzyme A was prominent in the RNA-Seq data and granzyme A deficient mice showed reduced arthritis, with no effects on viral loads. Arthritic disease could also be ameliorated in wild-type mice with a granzyme A inhibitor. Elevated circulating granzyme A levels were seen in non-human primates infected with CHIKV and in human CHIKV patients. Granzyme A thus emerges to be a major driver of CHIKV-mediated arthritic inflammation and a potential target for anti-inflammatory interventions.

## Introduction

Chikungunya virus (CHIKV) belongs to a group of mosquito-borne arthritogenic alphaviruses that include the primarily Australian Ross River and Barmah Forest viruses, the African o’nyong-nyong virus, the Sindbis group of viruses and the South American Mayaro virus [1]. The largest documented outbreak of CHIKV disease ever recorded began in 2004 in Africa and spread across the Indian Ocean to Asia, east to Papua New Guinea and several pacific islands, with small outbreaks also seen in Europe. In late 2013 the epidemic reached the Americas, spreading through the Caribbean, Central and South America, with autochthonous transmission also reported in the USA [2,3]. Millions of cases have been reported. Symptomatic infection of adults with CHIKV is nearly always associated with acute and often chronic polyarthralgia and/or polyarthritis, which can be debilitating and usually lasts weeks to months, occasionally longer [1,4]. At present, no particularly effective drug or licensed vaccine is available for human use for any of these alphaviruses; although paracetamol/acetaminophen and non-steroidal anti-inflammatory drugs can provide relief from rheumatic symptoms [1,5] and CHIKV vaccines are in development [6,7].

CHIKV infection usually results in a 5–7 day viraemia, which is primarily controlled by a rapid type I IFN response [8–11] and subsequently by anti-viral antibodies [12–15]. Infection also drives a pro-inflammatory response with the up-regulation of multiple inflammatory mediators [16–24]. CHIKV arthropathy is generally viewed as an immunopathology [13,25,26], with the pro-inflammatory arthritogenic response sharing similarities with rheumatoid arthritis [27]. The arthritogenic response is triggered by viral infection of joint tissues and is associated with a robust mononuclear cell infiltrate comprised primarily of monocytes, macrophages, NK cells and some T cells [28,29]. An important role for CD4 T cells in driving CHIKV arthritis has been established [27,30], although the role of IFNγ is less clear [27,30,31].

A major burden of CHIKV disease is chronic or persistent polyarthralgia/polyarthritis [4,32], with the evidence currently suggesting that such ongoing arthritic disease is due to persistence of virus and/or viral material in joint tissues [13,20,33]. Whether such viral material (i) represents replicating virus or replicating viral RNA [13] with mutations that promote persistence [34,35] or (ii) simply represents delayed clearance of non-replicating viral material [2], remains unclear. Whether chronic rheumatic disease is associated with the development of new inflammatory processes (distinct from those prominent during the acute phase) is also unclear.

We have developed an adult C57BL/6J (wild-type) mouse model of acute and chronic CHIKV infection and arthritis that recapitulates many aspects of human disease [13,28]. The model has been widely adopted for testing new interventions [25,36–43], although how well the mouse recapitulates the full spectrum of inflammatory responses seen in humans remains unclear.

A key goal of CHIKV arthritis research is to identify potential new targets for anti-inflammatory drug interventions to improve treatment options for CHIKV arthritis [25,26] and perhaps related diseases [44]. Such interventions should clearly neither compromise anti-viral immunity [25,45] nor trigger other immunopathologies [46]. Herein we describe an RNA-Seq study of lymph nodes and feet in the adult wild-type mouse model of CHIKV infection. The study was undertaken to explore in depth the anti-viral and pro-inflammatory responses in acute and chronic infection, and to identify new players in arthritic inflammation.

## Results

### Transcriptional profiling of CHIKV infection in adult wild-type mice

We undertook transcriptional profiling of whole hind feet and inguinal lymph nodes using the previously described adult wild-type mice model of acute and chronic CHIKV infection and arthritic disease [13,28]. Poly-adenylated RNA from whole hind feet (days 2, 7 and 30 post infection) and lymph nodes (days 2 and 7 post infection) from infected mice, and from feet and lymph nodes of mock infected mice were analyzed by RNA-Seq. Day 2 represents the day of peak viraemia, day 7 acute arthritis [28], with day 30 representing chronic arthritic disease [13]. Three biological replicates, each comprising pooled RNA from 4 mice, were sequenced using 3 lanes of the Illumina HiSeq 2000 platform. Quality control analyses and read alignment data are shown in S1 Fig. The Tuxedo pipeline was used to identify differentially expressed genes (DEGs) in the infected tissues at the different times post infection compared to mock infected controls. The DEG lists (where q<0.01 and fold change >2) and the up and down-regulated genes (with the additional filter of FPKM > 1 in at least one sample) are provided in S1 Table.

### Concordance with human inflammatory gene expression

The genes and/or proteins reported to be up-regulated in previous studies on CHIKV patients were all identified in this RNA-Seq analysis of mouse tissues (Table 1). Most of these genes/proteins are associated with inflammation (Table 1), suggesting a good concordance in pro-inflammatory gene expression in this mouse model and in human patients following CHIKV infection.

**Table 1:** Concordance of up-regulated genes identified by RNA-Seq in the current study of CHIKV infected mice and previously published protein and mRNA expression studies in human CHIKV patients. For studies showing protein expression of mouse mediators see S2 Table.

Genes		RNA-Seq in mice			Human CHIKV studies		
		Day 2	Day 7	Day 30	Acute	Chronic	*In vitro*
Cytokines	IFNα	Ft (▲^2^)	USR (+6.1)	USR (+5.2)	[16–19]^1^	PBMC & synovium [20]	
		USR (6.3)			PBMC [20]		
	IFNβ	Ft (▲^2^)	USR (+7.7)	USR (+5.3)			[47]
		USR (6.16)					
		LN (▲^2^)					
	IFNγ	LN (▲10.7)	Ft (▲211) ^3^	Ft (▲21.6) ^3^	[16–19,22]^1^	[21]^1^	
		USR (6.04)	USR (5.0)	USR (3.8)	T cells [48]	[48]	
	IL1β	Ft (▲2.4)	Ft (▲3.7)	Ft (▲1.4)	[16]^1^		[47]
		LN (▲2.4)		USR (+6.3)			
	IL2	Ft (▲^2^)	Ft (▲^2^)	USR (+4.78)	[16–20,23,24]^1^		
	IL4	Ft (▲1.47)	LN (▲2.81)	USR (+0.75)	[16–20,24]^1^		
	IL6	LN (▲14.4)	Ft (▲2.2)	Ft (▲2.2)		[18,21]^1^	[47]
		Ft (▲22.8)		USR (+4.7)		Synovial fluid [20]	
	IL7	USR (+3.2)	USR (+4.4)	USR (+2.8)	[16–19,24]^1^		
	IL10	Ft (▲16.4)	LN (▲2.2)	Ft (▲3.9)	[16–20,22–24]^1^	Synovium [20]^1^	
			Ft (▲30.1)				
	IL12	USR (+5.6)	USR (+6.5)	USR (+2.7)	[16–18,20,23,24]^1^	[20]	
	IL15	Ft (▲5.2)	Ft (▲2.3)	Ft (▲1.9)	[16–19] ^1^		
	IL17	USR (+6.0)	USR (+4.7)	USR (+4.8)	[16–19,23,24,49]^1^		
	IL18	USR (+5.5)	USR (+5.8)	USR (+4.2)	[19,50]^1^		
	IL18bp	Ft (▲4.76)	Ft (▲7.53)	NS	[50]^1^		
	TNFα	Ft (▲4.29)	Ft (▲7.89)	Ft (▲1.8)	[16,20,21,23,24]^1^	[18,20,21]^1^	
				USR (+8.4)			
	G-CSF	Ft (▲4.02)	NS	NS	[16–19,24]		
		LN (▲6.12)					
**Chemokines**	CCL1	Ft (▲1.58)	Ft (▲1.67)	Ft (▲ 3.47)		PBMC[20]	
	CCL2	Ft (▲36.6)	Ft (▲21.6)	Ft (▲3.3)	[16–19,22–24,49]^1^	Synovial fluid [20]	
		LN (▲6.7)					
	CCL3	Ft (▲16.6)	Ft (▲11.7)	Ft (▲3.1)	[16–19]^1^	PBMC[20]	[47]
		LN (▲7.0)	LN (▲10.7)				
	CCL4	Ft (▲56.6)	Ft (▲37.4)	Ft (▲4.6)	[16–19,24]^1^	PBMC[20]	
		LN (▲10.0)					
	CCL7	Ft (▲29.75)	Ft (▲23.4)	Ft (▲ 4.38)		PBMC[20]	
		LN (▲6.47)					
	CCL11	Ft (▲3.75)	Ft (▲2.02)	USR (+1.71)		Sera[18]	
	CCL19	Ft (▲5.17)	Ft (▲3.44)	NS		PBMC[20]	
	CXCL1	Ft (▲5.08)	Ft (▲1.97)	Ft (▲3.02)		PBMC[20]	
		LN (▲2.13)					
	CXCL2	Ft (▲2.38)	NS	NS		PBMC[20]	
		LN (▲3.93)					
	CXCL3	Ft (▲4.28)	Ft (▲4.09)	Ft (▲2.86)		PBMC[20]	
	CXCL5	Ft (▲2.66)	Ft (▲2.39)	Ft (▲1.68)		PBMC[20]	
		LN (▲2.16)	LN (▲1.68)				
	CXCL8 (IL8)	USR (2.0)	USR (2.65)	(USR (2.76)	[18,20]	PBMC[20]	
	CXCL9	Ft (▲391.5)	Ft (▲322.4)	Ft (▲62.1)	[16–19,22,23]^1^	PBMC[20]	[47]
		LN (▲11.1)					
	CXCL10	Ft (▲869.2)	Ft (▲185.0)	Ft (▲31.4)	[16–19,22,23]^1^	[51]	[47]
		LN (▲16.0)					
	CXCL11	Ft (▲116) LN (▲125)	Ft (▲51.68)	Ft (▲2.08)		PBMC[20]	
**Receptors**	CCR1	Ft (▲1.7)	Ft (▲4.12)	Ft (▲1.93)		PBMC[20]	
		LN (▲1.7)					
	CCR2	NS	Ft (▲5.69)	Ft (▲3.98)		PBMC[20]	
	CCR3	NS	NS	Ft (▲2.13)		PBMC[20]	
	CCR5	Ft (▲9.24)	Ft (▲33.6)	Ft (▲14.9)		PBMC[20]	
		LN (▲3.26)					
	CCR8	NS	Ft (▲2.44)	Ft (▲1.34)		PBMC[20]	
	CCRL2	Ft (▲15.4)	Ft (▲3.76)	NS		PBMC[20]	
		LN (▲7.58)					
	CXCR6	NS	Ft (▲5.85)	Ft (▲2.98)		PBMC[20]	
	CYFIP2	NS	Ft (▲1.99)	Ft (▲1.44)		PBMC[20]	
	IL1RA	Ft (▲3.49)	Ft (▲2.48)	NS	[16–19,22]^1^		
		LN (▲18.6)					
	PARP-1	USR (407)	USR (+4.5)	USR (+4.2)		Synovium [20]	
**Others**	CASP1	Ft (▲2.50)	Ft (▲3.0)	NS			[47]
	FGFβ	USR (+2.6)	USR (+1.7)	USR (+1.8)	[16–19]^1^		
	MMP2	NS	NS	Ft (▲1.57)		PBMC[20]	
	MYD88	Ft (▲1.89)	Ft (▲3.33)	NS		PBMC[20]	
		LN (▲3.71)					
	PTX3	Ft (▲18.27)	Ft (▲2.56)	NS	[52]		

Lymph node (LN); Feet (Ft); Fold change (▲); Upstream regulator (USR) with activation z-score; not significant (NS).

^1^ from human sera/plasma samples.

^2^ infinite fold change (nominally given a value of log_2_ of 21 in S1 Table).

^3^ FPKM<1.

Many of the up-regulated genes identified in this RNA-Seq analysis have also been shown to be up-regulated (at the gene and/or protein level) in previously published mouse and monkey studies (S2 Table).

### Global gene expression patterns

**Fig 1:**
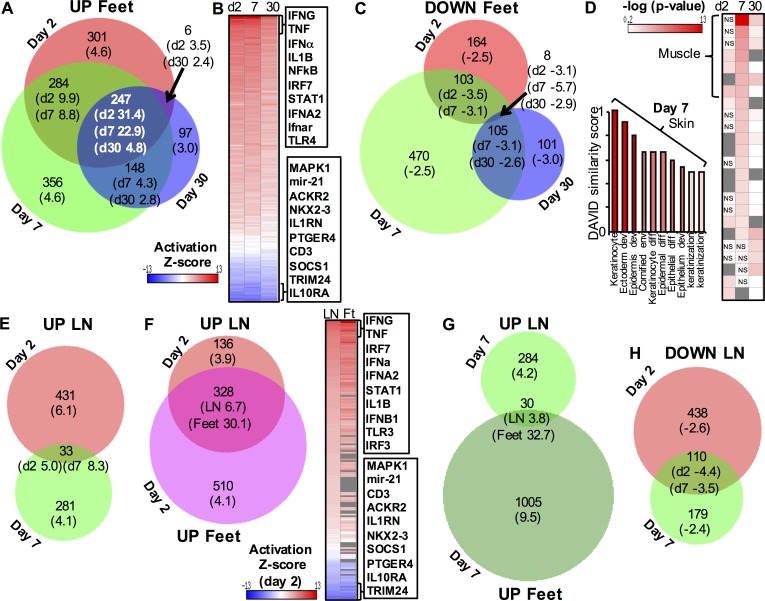
Global analysis of gene expression. (A) Venn diagram [70] of up-regulated genes in feet. Only genes with q<0.01, fold change >2 and FPKM >1 were included. Numbers in parenthesis represent the mean fold change of all the genes in the segment; note genes which are shared between all time points show the highest mean fold changes. (B) Heat map of−log_10_ z scores from Ingenuity upstream regulator analysis of the genes shown in A. Scores are ranked highest to lowest (mean of the 3 time points). The top and bottom 10 upstream regulators are shown on the figure; the full data set is shown in S3 Table. (C) Venn diagram of down-regulated genes in feet. Numbers in parenthesis represent the mean fold change. (D) Heat map of p values from Ingenuity canonical pathway analysis of the genes shown in C, with day 7 pathways ranked most to least significant. A DAVID analysis of the same genes (bar chart) showing similarity scores. The full data sets for these figures are provided in S3 Table. (E) Venn diagram of up-regulated genes in lymph nodes on days 2 and 7. (F) Venn diagram of up-regulated genes on day 2 in lymph nodes (LN) and feet (Ft); and heat map of−log_10_ z scores from Ingenuity upstream regulator analysis of these genes. The first column of the heat map shows z scores from day 2 lymph nodes ranked highest to lowest (the full data set is provided in S3 Table). (G) Venn diagram of up-regulated genes in lymph nodes on days 7 and feet on day 7. (H) Venn diagram of down-regulated genes in lymph nodes on days 2 and 7.

Venn diagram presentation of the up-regulated genes in feet illustrated that many up-regulated genes were shared between days 2, 7 and 30, with these shared genes also showing the highest mean fold change (Fig 1A). These 247 shared genes (fold change >2, FPKM>1, q<0.01, S3 Table) were overwhelmingly type I IRGs (as defined by Interferome [53]) and contained many anti-viral effectors, some of which have previously been described in alphavirus studies, such as Mx1 [54], viperin [55], ISG15 [56] and Ifit1 [57] (Table 2). Sensing and signaling proteins were also prominent and included IRF7 [9], Usp18 [58], Stat1, IRF1, IRF5 and IRF8. Tmem731 (STING) [59] (Table 2) and Trex1 [60] (S1 Table) were up-regulated, although the mechanisms and implications remain to be established [61–63]. CXCL10 was the most up-regulated chemokine (Table 2), with only some chemokines, such as CCL2, well studied in alphavirus infections [33,46]. As might be expected, complement [64], immunoproteasome genes and T cell response associated genes [27,30] were present (Table 2). Although granzyme B up-regulation has been noted previously [65], its prominence (Table 2, Gzmb) was perhaps unexpected given the limited role played by cytotoxic T cells and NK cells in protection against alphavirus infections [13,30,65,66]. Also prominent were interferon-inducible guanylate binding proteins, immunity related GTPases [67], C-type lectins and membrane-spanning 4-domains subfamily A (Ms4a) genes (Table 2), which have not been extensively studied in alphavirus infections. Most of the genes in the latter two groups and Cd300a, recently reported as a virus attachment factor [68], are expressed by monocytes/macrophages [69], which dominate the CHIKV inflammatory infiltrate.

**Table 2:** Grouping of selected genes from the 247 shared up-regulated genes in feet.

	Day 2	Day 7	Day 30		Day 2	Day 7	Day 30
**Effectors**				**Chemokines**			
Mx1 (MxA)	339.9	29.8	4.4	Cxcl10	869.2	185.0	31.4
Iigp1	250.0	72.4	15.3	Cxcl9	391.5	322.4	62.0
Isg15	226.8	44.6	4.9	Ccl5	62.8	41.3	9.3
Rsad2 (Viperin)	221.1	15.5	4.4	Ccl4	56.6	37.4	4.6
Ifi44	215.5	44.8	10.6	Ccl2	36.7	21.6	3.3
Ifit1	204.6	24.8	7.8	Ccl7	29.8	23.5	4.4
Ifit3	125.2	23.1	5.8	Ccl12	29.3	27.1	9.8
Mx2 (MxB)	111.7	9.6	2.3	Ccl3	16.6	10.0	3.1
Ifit2 (ISG54)	75.6	10.8	2.7	Ccr5	9.2	33.6	14.9
Zbp1 (DAI)	49.7	59.0	11.8	Ccr7	7.1	10.8	2.2
Oas2	29.2	15.0	3.4	Ccl8	3.9	9.9	2.6
Samhd1	10.3	9.5	3.5	Cxcl16	2.1	5.0	3.0
Apobec3	5.1	7.9	2.1				
Apobec1	3.7	8.2	3.3	**Complement**			
Rnase6	2.6	5.7	3.6	C2	7.4	3.9	2.2
				C3ar1	2.4	7.1	4.1
**Sensing/Signalling**							
Oasl1	152.1	37.1	5.2	**Immunoproteasome**			
Irf7	116.2	38.8	4.2	Psmb8	12.9	14.9	4.6
Apol9b	68.1	19.1	7.6	Psmb9	12.1	14.8	4.4
Usp18	62.0	10.4	3.2	Psmb10	10.9	10.7	2.7
Apol9a	59.6	19.1	9.6				
Oasl2	54.0	20.6	5.5	**Inflammasome**			
Stat1	22.5	18.6	2.8	Pyhin1	26.8	22.8	4.3
PARP9	17.3	7.0	2.4	Nlrc5	18.9	12.8	4.0
DTX3L	15.8	7.5	2.2	Nlrp3	3.4	7.3	3.1
Tlr9	11.2	17.5	3.3	Naip2	2.3	4.6	2.6
Irf1	9.8	13.6	2.9				
Trim14	8.8	5.3	2.2	**Guanylate binding proteins (p65 GTPases)**			
Tlr2	6.8	3.7	3.1	Gbp5	79.0	44.4	8.3
Trim12c	5.6	3.4	2.0	Gbp2	63.5	53.5	13.0
Traf1	4.8	6.3	3.2	Gbp3	45.3	29.2	7.7
Tmem173	3.7	6.2	2.2	Gbp7	33.3	16.5	5.2
Tlr13	3.6	10.6	4.4	Gbp6	28.6	28.8	2.6
Irf8	3.3	9.4	3.0	Gbp8	11.5	52.5	8.0
Irf5	2.8	4.8	2.0				
				**Immunity related GTPases**			
**T cell associated**				Mx genes (see above)			
Gzmb	67.5	623.4	18.3	Gm4841 (Ifgga3)	686.9	322.9	10.8
H2Q6	27.9	17.6	7.7	Gm12185	269.2	183.7	13.2
H2Q7	20.3	17.6	7.5	Iigp1	250.0	72.4	15.3
H2T24	18.5	17.6	3.9	Gm12250	130.9	129.0	13.8
H2Q4	16.1	17.6	4.7	Tgtp1	76.4	88.0	8.3
H2Q8	14.1	17.6	4.4	Igtp	64.2	45.5	6.4
Tap1	13.0	11.4	3.2	Gm4951 (Ifgga2)	46.7	39.2	7.1
H2T10	11.5	17.6	3.0	Irgm1	39.4	26.9	3.9
H2T23	9.8	17.6	3.0	Irgm2	37.7	23.2	5.0
H2K1	7.6	17.6	4.0				
B2m	7.3	8.7	4.2	**C-type lectins**			
Hck	6.8	19.2	4.5	Clec4e	10.5	14.1	12.0
Tapbp	6.8	4.8	2.2	Clec4d	3.0	4.5	9.4
Tap2	6.3	3.7	2.3	Clec4a3	2.9	9.5	3.1
H2M3	5.9	17.6	4.4	Clec4a1	2.5	8.7	3.2
H2D1	4.9	17.6	4.0	Clec12a	2.1	11.4	7.0
Cd53	3.0	5.8	2.9	Clec7a	2.0	5.6	3.4
Lck	2.6	12.3	3.2				
Ccdc88b	2.5	7.4	2.2	**Membrane-spanning 4-domains**			
Lyn	2.3	5.7	2.7	**subfamily A member**			
Tagap	2.0	3.4	2.1	Ms4a4c	65.8	67.0	5.1
				Ms4a4b	9.5	39.3	6.1
**Virus attachment factor**				Ms4a6c	5.3	13.5	3.7
Cd300a	2.8	6.6	2.7	Ms4a6d	5.0	10.2	3.4
				Ms4a6b	4.7	10.3	2.8
				Ms4a8a	4.6	7.1	2.6

Refer to Fig 1A for derivation of the 247 genes. Mean fold change for days 2, 7 and 30 are shown. The full gene list is provided in S3 Table (feet shared).

Ingenuity pathway analysis (IPA) of up-regulated genes illustrated a high degree of similarity in the upstream regulators (direct and indirect) identified at the 3 time points in feet (Fig 1B). IPA canonical pathway analysis also showed considerable overlap (with many pathways associated with T cells) (S2 Fig). Gene induction profiles and inflammatory pathways were therefore surprisingly similar despite the different stages of infection and disease; day 2 (peak viraemia), day 7 (acute arthritis, no viraemia) and day 30 (chronic disease, persistent viral RNA) [13,28]. IPA analysis of genes uniquely up-regulated on day 30 (i) identified pathways already identified for days 2 and 7 and (ii) showed that many of the genes were associated with tissue repair (using the IPA Diseases & Functions feature).

For the down-regulated genes in feet, a large number of genes were uniquely down-regulated on day 7 (Fig 1C). IPA canonical pathway analysis also showed minimal overlap in pathways between the 3 time points (Fig 1D, heat map; S3 Table). These analyses and the low mean fold change (-2.5) suggest that a major influence on this data set is the pronounced cellular infiltration seen on day 7 [28], which would effectively dilute (and down-regulate) the mRNA of resident cells. This contention is supported by the observation that the top 10 terms identified by DAVID (v6.7) functional gene annotation analysis were associated with keratinocytes (Fig 1D, bar chart; S3 Table), cells that are not a major target of infection in C57BL/6 mice [9]. The top IPA canonical pathways for day 7 were associated with muscle (Fig 1D, heat map; S3 Table), with both the dilution effect and viral infection [9,71] likely responsible.

In contrast to feet, DEGs up-regulated in lymph nodes showed only minimal overlap between days 2 and 7 (Fig 1E). However, up-regulated genes in lymph nodes on day 2 showed a considerable overlap with genes up-regulated in feet on day 2 (Fig 1F, Venn diagram). IPA upstream regulator analysis also showed a high degree of concordance between pathways in lymph nodes and feet on day 2 (Fig 1F, heat map). This likely reflects the systemic nature of the infection and argues that early innate responses are not overly tissue specific.

Up-regulated genes on day 7 in lymph nodes, as might be expected, were dominated by immunogobulin genes, which represent 60% of the top 150 genes (S1 Table). The top terms from a DAVID functional gene annotation analysis were associated with cell division, consistent with the expected proliferation of B and T cells. In contrast with day 2 (Fig 1F), there was minimal overlap between up-regulated genes in lymph nodes and feet on day 7 (Fig 1G). By the time adaptive immune responses are developing and arthritis is peaking, the infiltrates in lymph nodes and arthritic feet thus appear to share relatively few genes.

The down-regulated genes from lymph nodes on days 2 and 7 showed some overlap (Fig 1D). IPA upstream regulator analysis also showed some overlap in pathways, with top pathways (as expected) generally indicative of immune activation.

### CHIKV genome sequence data

**Fig 2:**
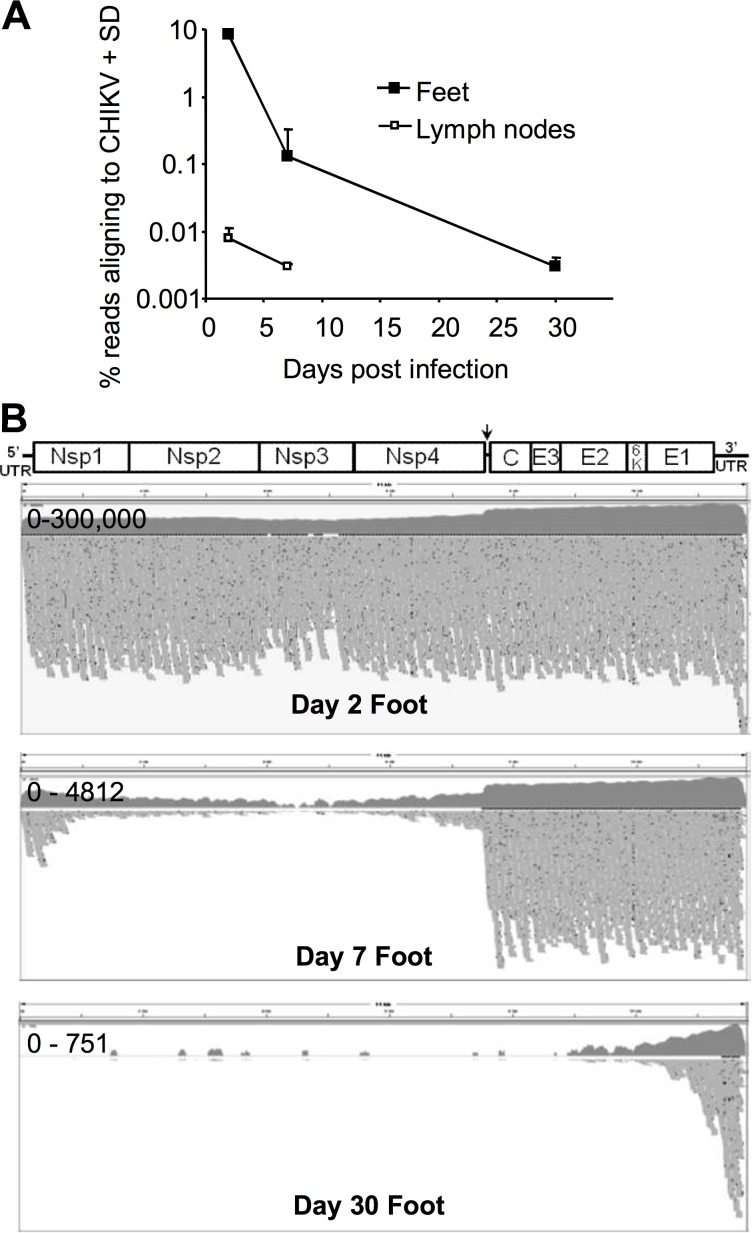
CHIKV genome. (A) The percentage of all the reads that aligned to the mouse genome for each tissue and time point. Read alignment numbers are provided in S1D Fig. (B) Examples of alignments of RNA-Seq reads from 3 foot samples mapped to the CHIKV genome (map quality threshold 20) viewed using Integrated Genomics Viewer (IGV version 2.3.34). The CHIKV genome is shown at the top for reference (arrow represents position of the sub-genomic promoter). Upper graphs (dark grey) show sequence coverage (log scale) for each nucleotide position in the CHIKV genome with y axis scale ranges (e.g. 0–300,000) shown in the left hand corners. The bottom graphs are “squished” views of the 100 bp reads aligned to the CHIKV genome (each grey horizontal bar represents one read). Black spots represent deletions/insertions within each read.

Reads that did not map to the mouse genome were mapped to the CHIKV genome (S1D Fig). For feet on day 2, >8% of all sequencing reads aligned to the CHIKV genome, with 84% of reads aligning to the mouse genome (Fig 2A, S1D Fig). The number of reads aligning to the CHIKV genome dropped to 0.003% by day 30 (Fig 2A, S1D Fig), a reduction consistent with previous qRT-PCR data [13]. Examples of read alignments to the CHIKV genome are shown in Fig 2B and S3A Fig. The higher sequence coverage for the structural genes (evident at all 3 time points), reflects the known higher levels of subgenomic 26S RNA (encoding C to E1) compared to genomic RNA present in alphavirus infected cells [34]. The 3′ bias in sequence coverage, clearly evident on day 30 (Fig 2B), may represent an artifact of the Illumina HiSeq sequencing platform [72].

Although the low fidelity RNA replication of CHIKV [73] might predict the rapid emergence of sequence variants, we were unable to identify any consistent or high frequency changes (S3B Fig). Although some changes were identified (i) for each nucleotide position the percentage of reads showing a different nucleotide to the reference sequence rarely exceeded 10%, (ii) nucleotide sites with >2% of reads showing changes from the reference sequence were associated with areas of low read coverage (S3B Fig) and (iii) some consistent deletions/insertions (present in up to 10% of reads) were associated with runs of identical polynucleotides. Changes above a background sequencing error rate of ≈2% thus appear largely to represent sequencing artifacts. The ratios of synonymous to non-synonymous mutations were also consistent with random changes (S3C Fig).

### Dominance of interferons and interferon regulated genes

**Fig 3:**
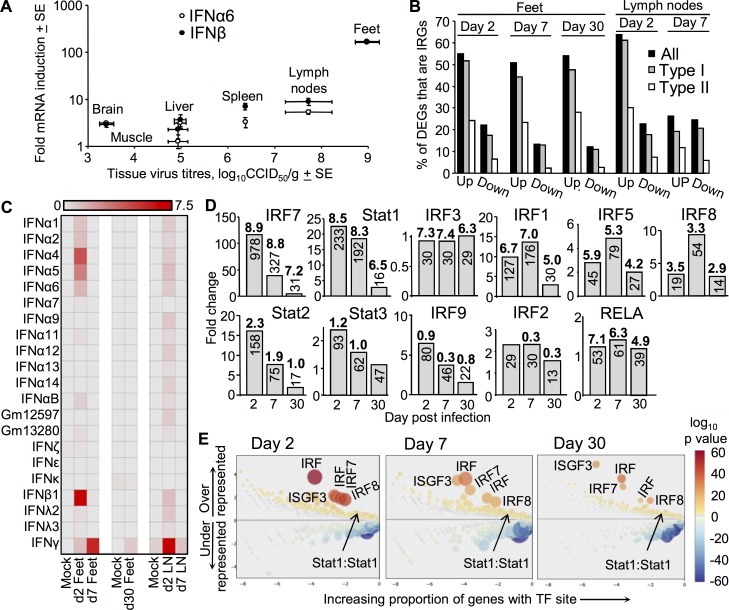
The interferon signature. (A) Expression of IFNα6 and IFNβ as determined by qRT-PCR in different tissues on day 2 post CHIKV infection (n = 3 mice), the time of the peak IFNα/β response [28]. Values are normalized to RPL13a mRNA levels and expressed as fold induction relative to mock infected controls (n = 3). CHIKV titres in the tissues were determined as described [28]. Spearman’s correlation tests showed a significant relationship between IFN mRNA levels and viral titres (IFNα6 Spearman’s rho = 0.829 p = 0.042, IFNβ Spearman’s rho = 0.943 p = 0.005). (B) For all samples the up- and down-regulated DEGs (for which FC>2, q<0.01 and FPKM>1, see S1 Table) were analyzed using Interferome and the percentage of these DEGs that are interferon regulated genes (IRGs) is shown. (Interferome does not distinguish between genes directly or indirectly stimulated by IFNs, and some type I and/or II IRGs may not be identified by Interferome). (C) Heat map of FPKM values for all IFN genes identified by the RNA-Seq analysis (the same data is plotted as bar chart in S4 Fig). (D) Transcription factors associated with IFN responses in feet. Fold change of indicated transcription factors with vertical numbers representing the mean FKPM values (for the 3 biological replicates). Horizontal bold numbers represent the activation Z scores for the indicated transcription factor as determined by the direct function of the upstream regulator analysis of IPA; corresponding p values are provided in S5 Fig. (E) CiiiDER analysis of putative transcription factor site enrichment in the up-regulated type II IRGs in feet (as identified by Interferome). Color and size of circles reflect p values of the enrichment. Calculations for x and y values and the input/output data for the labeled transcription factors are provided in S7 Fig.

The importance of the type I interferon (IFN) response for protection against lethal CHIKV infection is well established [9–11]. The upstream regulator analysis also showed that many of the top upstream regulators were associated with the type I IFN response (Fig 1B). qRT-PCR analysis illustrated a good correlation between IFNβ or IFNα6 mRNA levels and the tissue CHIKV titers, with feet and lymph nodes (the tissues analyzed by RNA-Seq in this study) showing the highest levels of both CHIKV and IFNβ/IFNα6 mRNA levels (Fig 3A). Type I IFN induction was thus more virus titer-dependent than tissue-dependent.

Interferome (v2.01) analysis of the DEGs identified by RNA-Seq (S1 Table) illustrated that about half of up-regulated genes (in all samples except day 7 lymph nodes), and 10–20% of down-regulated genes, were type I IFN regulated genes (IRGs) (Fig 3B). In addition, 10–30% of up-regulated genes in all samples were identified as genes regulated by IFNγ (type II IRGs) (Fig 3B). This analysis provides the first quantitative assessment of the very considerable dominance of IFN responses, particularly the type I IFN response, during both acute and chronic CHIKV infection.

The RNA-Seq analysis provided the first detailed picture of all the type I IFN genes induced after CHIKV infection, with IFNβ and α4 dominating (Fig 2C and S4 Fig). The surprising observation (given Fig 3B) was the overall low abundance of type I IFN transcripts, which did not exceed an FPKM = 7 and was often close to FPKM = 1 (Fig 3C, S4 Fig), a frequently used cut-off for expression analyses [74,75]. Low abundance of type I IFN mRNAs may also explain why reporter mice expressing GFP from IFNα or IFNβ promoters [76] express undetectable levels of GFP after CHIKV infection [77]. These results suggest high bioactivity for type I IFN proteins and/or highly efficient translation of type I IFN mRNAs [9–11]. Despite persistence of viral RNA (Fig 2A), by day 7 and 30 type I IFN mRNA levels had dropped to background levels (Fig 3C, S4 Fig).

### Transcription factor analyses

The continued dominance of type I IRGs on days 7 and 30 (Fig 2C) despite the loss of significant type I IFN mRNA induction (Fig 2B, S4 Fig), argues that type I IFN-independent induction of type I IRGs (although well described [63,78,79]) essentially takes over after the brief period of type I IFN production. To better understand this process, an examination of transcription factor usage was undertaken. The direct upstream regulator function of IPA identified IRF7, STAT1, IRF3, IRF1 and IRF5 [80] in the top 10 upstream regulators for each time point (ranked by activation Z scores), with these transcription factors also showing (i) high fold change (with the exception of IRF3) and (ii) high FPKM (mRNA expression) values (Fig 3D). Other transcription factors identified by this analysis were IRF8 [81], Stat3 [82], IRF1 [83,84], IRF2 [85], and Stat2/IRF9 (with unphosphorylated ISGF3 able to signal [86]) (Fig 3D, S5A Fig). RELA was also identified in the top 10 (ranked by activation Z scores), but was only marginally up-regulated (Fig 3D, S5A Fig). These results were supported by a transcription factor site analysis using a new program (CiiiDER, Gearing et al, in prep) (S5B Fig). Although identification of IRF7 and IRF3 would be expected [9,78,87]; the role(s) of the other transcription factors identified herein remain to be fully explored in alphaviral infections [79,81,83,84,86].

### The role of IFNγ

The accumulated data might suggest IFNγ plays an important role in CHIKV infections [28] (Figs 1B and 3B–3D, S4 and S5B Figs.), both to promote inflammation [27,31] and to mediate anti-viral activity [88–90]. However, CHIKV infection of IFNγ^-/-^ mice led to only a slightly elevated/extended RNAemia [30] or viraemia (S6A Fig), and only a marginal decrease in arthritic disease (S6B Fig), which was largely due to a reduction in edema (S6C and S6D Fig).

The limited effects of IFNγ deficiency prompted an analysis of putative transcription factor sites in the promoters of the type II IRGs up-regulated in feet (white bars, Fig 3B) using the CiiiDER program. Contrary to expectations, putative IRF7, ISGF3, IRF8 and consensus IRF sites were significantly over-represented in these genes (Fig 3E; formulas for calculating x and y values and the analysis inputs and outputs are provided in S7 Fig). Putative Stat1:Stat1 sites, although present in ≈45% of the type II IRGs, were also present in ≈34% of background genes thereby reducing significance scores (Fig 3E, S7 Fig). The mild phenotype in IFNγ^-/-^ mice might thus be explained by redundancy in the induction of type II IRGs. The reverse, a compensatory role for IFNγ in the absence of IFNα/β has also previously been suggested [91].

### Prominence of granzymes A, B and K in the RNA-Seq data

**Fig 4:**
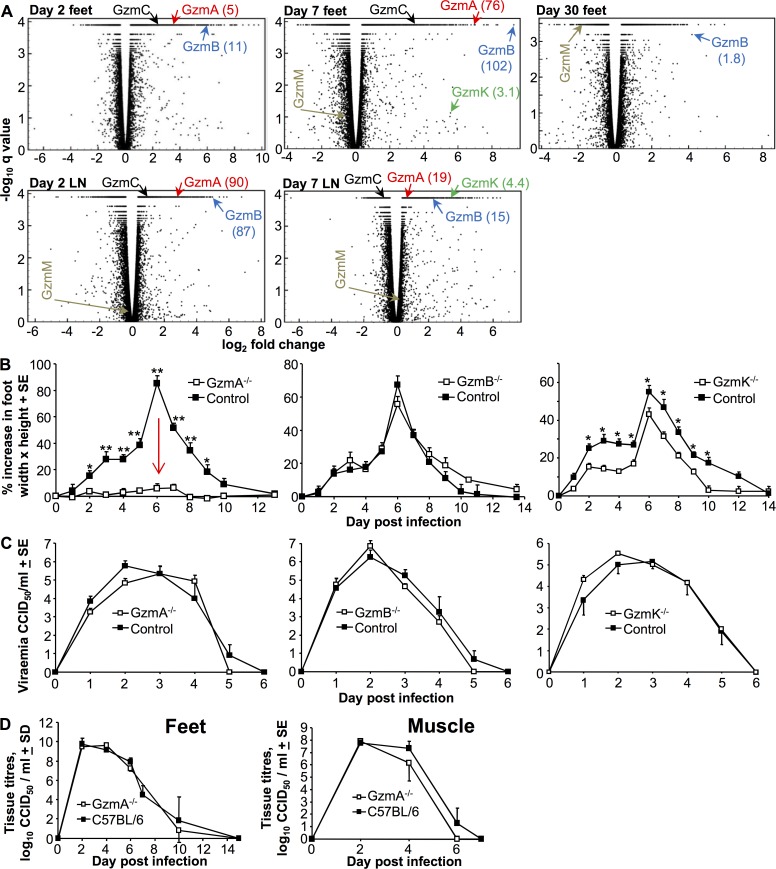
Granzyme gene expression and CHIKV infection in granzyme deficient mice. (A) Volcano plots of host gene expression from the RNA-Seq analysis of feet and lymph nodes (LN) at the indicated day post infection. Only genes where FPKM ≥1 in at least one sample in the pair wise comparisons were included in the plots. Positions of the granzyme (Gzm) genes are indicated by arrows, with values in parenthesis representing FPKM values. (B) Foot swelling in granzyme deficient mice. Mice were infected as above and foot swelling monitored. GzmA^-/-^ mice; * p<0.03, ** p<0.001 (Kolmogorov-Smirnov tests, n = 8–10 mice per group). GzmK^-/-^ mice; *p<0.031 (Mann Whitney U tests, mean of two independent experiments is shown, n = 12–14 per group). (C) Viraemia in granzyme deficient mice. Granzyme deficient mice were infected with CHIKV and the viraema measure on the indicated days. No significant differences in viraema were apparent: GzmA^-/-^ vs C57BL/6 controls (n = 6 mice per group); GzmB^-/-^ vs C57BL/6 controls (n = 8–10 mice per group); GzmK^-/-^ vs C57BL/6 controls (n = 12–14 mice per group). (D) Tissue CHIKV titers in GzmA^-/-^ and C57BL/6 mice. Feet; n = 6–12 GzmA^-/-^ and n = 12–20 C57BL/6 feet per time point; data obtained from 2 independent experiments. Muscle; n = 3–6 mice per time point.

An important objective of the RNA-Seq analysis was to identify new players in arthritic inflammation that may present new targets for intervention. Interrogation of the data revealed that granzyme A, B and K often show highly significant induction, high fold change, and for granzyme A and B, high FKPM values (Fig 4, Table 2). These granzymes are classically associated with cytolytic activities and their expression and secretion by cytotoxic T cells and NK cells is well described [92–95]. However, granzymes (particularly A and K) have also been associated with promoting inflammation in a number of settings [92–94,96,97].

### CHIKV infection in granzyme deficient mice

To assess the role of granzymes A, B and K in CHIKV infection and disease, mice deficient in these proteases (GzmA^-/-^, GzmB^-/-^ and GzmK^-/-^ mice) were infected with CHIKV. Strikingly, GzmA^-/-^ mice showed a dramatic reduction in foot swelling (Fig 4B). An independent repeat experiment with similar results is shown in (S8A Fig). No significant effect on foot swelling was evident in GzmB^-/-^ mice, but GzmK^-/-^ mice showed a significant, but less dramatic, reduction in foot swelling (Fig 4B).

**Fig 5:**
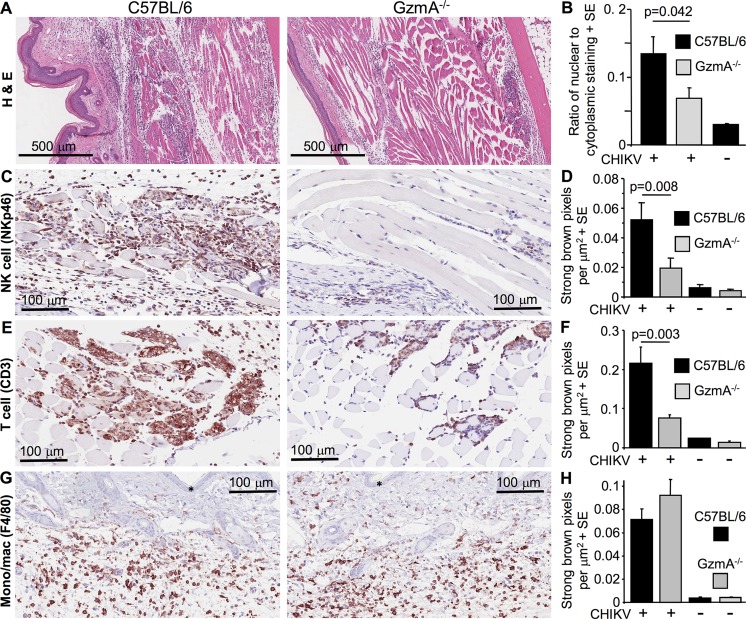
Histology and immunohistochemistry of GzmA^-/-^ mice feet. (A) H&E staining of feet on day 6 post infection in C57BL/6 and GzmA^-/-^ mice. Cellular infiltrates are characterized by high densities of blue staining nuclei; areas with pronounced cellular infiltrates are indicated by white oval outlines. (B) Aperio Positive Pixel Count determination of the ratio of blue (nuclear) to red (cytoplasmic) staining areas in whole foot sections day 6 post infection. Leukocytes tend to have a higher nuclear/cytoplasmic area ratio, so elevated ratios reflect increased leukocyte infiltrates [13]; (n = 6 feet from 6 mice per group, 3 sections per foot; statistics by 2 way ANOVA including a term for section). (C) Immunohistochemical staining for NK cells (anti-CD335/NKp46) clearly visible (brown staining) in muscle tissue of feet from CHIKV-infected C57BL/6 mice 6 days post infection (left). NK cell staining was less pronounced in GzmA^-/-^ mice (right). Blue counter staining with haematoxylin. (D) Aperio Positive Pixel Count determination of NK cell staining; strong brown pixels per μm^2^ in whole feet sections (3 sections per foot; n = 11–12 feet from 11–12 mice per group from 2 independent experiments. Statistics by Mann Whitney U test). (E) As for C for T cell (anti-CD3) staining. (F) As for D for T cell staining. Statistics by Kolmogrov Smirnov test. (G) As for C for monocyte/macrophage (F4/80) staining, which was prominent in subcutaneous tissues (* indicates epidermis). (H) As for D for monocyte/macrophage staining.

None of the granzyme deficient mice showed significant changes in the viraemia (Fig 4C), consistent with the general view that controlling the viraemia of cytopathic viruses (such as alphaviruses) is not overly reliant on T cell- or NK cell-dependent cytolytic activities [65,66,98]. Granzyme A deficiency has been associated with a failure to clear certain viral infections [99,100]; however, feet tissue titers were not significantly affected in GzmA^-/-^ mice (Fig 4D, Feet). In addition, the level of persistent CHIKV RNA in feet on day 30 post infection was not increased in GzmA^-/-^ mice when compared with C57BL/6 mice (S8B Fig). (In both humans and C57BL/6 mice, viral RNA persists for extended periods and is associated with chronic arthritic disease [13,20]). Cytotoxic T cells have been reported to be important for clearing alphavirus from muscle tissues in certain settings [101]; however, muscle tissue titers were also not significantly different in GzmA^-/-^ mice (Fig 4D, Muscle). The reduced inflammation in GzmA^-/-^ mice (Fig 4B and Fig 5) was thus not due to an effect of granzyme A deficiency on virus levels in inflamed tissues.

GzmA^-/-^ mice did not show any significant differences from C57BL/6 mice in their CHIKV-specific IgG2c and IgG1 responses (S8C Fig), indicating that anti-CHIKV antibody responses and the Th1/Th2 balance [102] were not significantly affected by granzyme A deficiency.

### Histology and immunohistochemistry of CHIKV infected GzmA^-/-^ mice

Histological examination of feet from GzmA^-/-^ mice showed that the densities of cellular infiltrates (a prominent feature of CHIKV arthritis [28]) were significantly reduced when compared with C57BL/6 mice (Fig 5A and 5B). This result is consistent with the reduction in foot swelling and supports the contention that granzyme A has a role in promoting arthritic inflammation.

Immunohistochemical analyses of whole foot sections from CHIKV-infected mice during peak arthritis illustrated that the densities of NK (Fig 5C and 5D) and T cells (Fig 5E and 5F), but not monocytes/macrophages (Fig 5G and 5H), was significantly reduced in GzmA^-/-^ when compared with C57BL/6 mice.

### Granzyme A inhibitor, Serpinb6b

**Fig 6:**
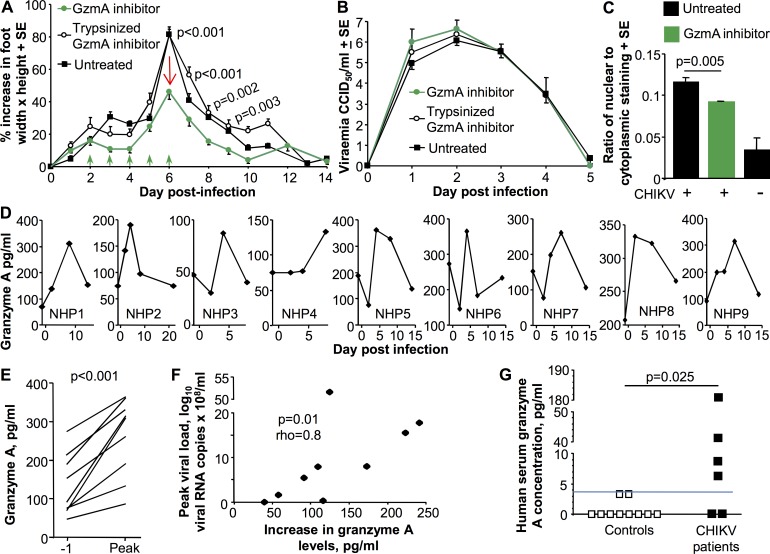
Granzyme A inhibitor and granzyme levels in CHIKV infected primates. (A) On days 2 to 6 post infection with CHIKV, C57BL/6 mice were treated i.v. with 10 μg of the granzyme inhibitor, Serpinb6b, or Trypsinized (inactivated) GzmA inhibitor (green arrows) or were left untreated. The data represents results from 2 independent experiments: GzmA inhibitor (n = 14 feet, 7 mice); Trypsinized GzmA inhibitor (n = 6 feet, 3 mice); untreated mice (n = 30 feet, 15 mice). Statistics by t test; p values provided for comparisons between granzyme A inhibitor and Trypsinized granzyme A inhibitor and are only provided where comparisons between granzyme A inhibitor and untreated mice were also significant. (B) Viraemia for the same mice as shown in A. (C) Aperio Positive Pixel Count determination of the ratio of blue (nuclear) to red (cytoplasmic) pixels in H&E stained whole foot sections day 6 post infection (as in Fig 5B). (n = 6 feet from 3 mice per group, 3 sections per foot; statistics by Kolmogorov Smirnov test). (D) Granzyme A levels in plasma samples from CHIKV-infected non-human primates (NHPs) measured using an ELISA kit. Data for 9 NHPs is shown; all NHPs had samples collected day -1, one day prior to CHIKV infection. (E) Granzyme A levels on day -1 and peak granzyme A levels plotted for the 9 NHPs. Statistics by paired t test. (F) Correlation between peak viral load (S9D Fig) and the increase in granzyme A levels from day -1 to peak (i.e. peak levels minus day -1 levels). Statistics by Spearman correlation. (G) Serum granzyme A levels in healthy controls and IgM positive symptomatic CHIKV patients. Granzyme A levels were determined using cytokine bead array and FACs. The limit of detection is deemed to be 3.7 pg/ml. Statistics by Kruskal-Wallis test.

The pro-inflammatory activity of granzyme A is believed to be due to its proteolytic activity [92,96,103,104], with extracellular or circulating granzyme A remaining proteolytically active [105,106]. Furthermore, a potent and specific endogenous inhibitor of mouse granzyme A has been identified, Serpinb6b [107]. To determine whether Serpinb6b might show therapeutic activity, C57BL/6 mice were injected i.v. with purified recombinant Serpinb6b [107] from day 2 to 6 post CHIKV infection. Treatment with this granzyme A inhibitor significantly reduced foot swelling (Fig 6A) without impacting the viraemia (Fig 6B). (Treatment was not associated with any noticeable side-effects during daily monitoring of mice). Proteolytic inactivation of Serpinb6b with trypsin reversed the anti-inflammatory activity back to that seen in untreated mice (Fig 6A). H&E staining also showed a reduction in the arthritic infiltrates in the feet of Serpinb6b treated mice (Fig 6C). These results support the view that granzyme A has an extracellular pro-inflammatory role in this setting, and that granzyme A represents a potential target for anti-inflammatory drugs.

### Evaluated plasma granzyme levels A in a non-human primate model of CHIKV infection

Elevated levels of circulating granzyme A have been detected in humans with a number of viral infections [105,108,109] or suffering from rheumatoid arthritis [110]. We have previously reported a non-human primate (NHP) model of CHIKV infection [33]. Using commercial ELISA kits, granzyme A and K levels were determined in plasma samples from such CHIKV-infected NHPs. In 9 out of 11 NHPs, plasma granzyme A levels increased relative to levels on day -1 (prior to infection) and usually peaked on day 4–8 post infection (Fig 6D). (The initial drop in granzyme A levels in NHPs 3, 5, 6 and 7 on day 2 coincides with the transient lymphopenia often seen at this time [33]). Taken as a group, the peak granzyme A levels for the 9 animals were significantly elevated when compared with levels prior to infection (day -1) (Fig 6E). Using data from all 11 animals, significance was retained (S9A Fig). In addition, when mean levels of granzyme A for all NHPs were plotted over time, a significant elevation was again evident (S9B Fig). A similar treatment of granzyme K data showed no significant elevation in mean circulating granzyme K levels (S9C Fig). This may in part reflect a sensitivity issue, as increases in circulating granzyme K levels after viral infections can be substantially more modest than those seen for granzyme A [111].

For most plasma samples in which granzyme levels were tested, viral loads were also measured; viral loads for all nine animals over time are shown in S9D Fig. No correlation between granzyme A level and viral load was apparent when using data from individual samples (S9E Fig). However, when peak viral loads (which occurred on day 2 post infection, S9D Fig) were plotted against the increase in granzyme A levels (i.e. the increase from day -1 to the peak) for each of the 9 NHPs in Fig 6D, a clear and significant positive correlation was observed (Fig 6F). Plotting peak viral loads against peak granzyme A levels also showed a significant correlation (S9F Fig). Thus the higher the viral load (the higher the disease severity [33] and) the higher the subsequent increase in circulating granzyme A levels.

### Evaluated circulating granzyme A levels in CHIKV patients

Serum levels of granzyme A were also measured in serum from control patients and a small cohort of deidentified symptomic CHIKV patients who had tested IgM positive for CHIKV. (IgM usually remains detectable by serology for 1–3 months [1]). The CHIKV patients showed significantly higher levels of serum granzyme A than controls (Fig 6F), suggesting that CHIK-infected humans, like NHPs, show elevated granzyme A levels after CHIKV infection.

## Discussion

Herein we describe the first detailed RNA-Seq analysis of CHIKV infection, covering the time of peak viraemia, and acute and chronic arthritis, in a widely adopted adult wild-type mouse model of CHIKV infection and disease [77]. The inflammatory mediators identified previously in CHIKV infected humans were also identified by this RNA-Seq analysis (Table 1), illustrating that the mouse model recapitulates known aspects of the human inflammatory response to CHIKV infection. This analysis also highlights the potential for using RNA-Seq data to provide a level of validation of mouse models in general.

The RNA-Seq analysis provided information on CHIKV genome expression and sequence. In feet up to 8% of all the reads from poly adenylated RNA mapped to the CHIKV genome (S1D Fig), attesting to the remarkably high replicative capacity of this virus [112]. We are unaware of any study suggesting such a high proportion of viral RNA to host mRNA *in vivo*, although it should be noted in this model CHIKV infection is via s.c. inoculation into the feet [28]. The persistence of CHIKV RNA in joint tissues seen herein is also consistent with previous reports of persistent CHIKV RNA in mice, monkeys and humans [13,20,33]. The notion that the viral genome might undergo adaptive changes to promote persistence [34,35,113] was not supported by the RNA-Seq analysis, with no consistent or abundant genomic changes identified. The lack of changes in persisting CHIKV RNA thus suggests the CHIKV RNA is either not replicating [2] or is replicating, but not adapting over time. Continuous viraemia in Rag1^-/-^ mice for 100 days also resulted in surprisingly few changes in the CHIKV genome [13].

Global expression profiles for feet and lymph nodes on day 2 (peak viraemia), feet day 7 (acute arthritis, no viraemia) and feet day 30 (chronic arthritis, persistent viral RNA) showed a surprisingly high level of overlap in both up-regulated genes and pathways, despite the differences in levels of infection, disease manifestations, immunity and tissues types [13,28]. This might in part be explained by the dominance of the IFN and inflammatory responses, which are largely independent of time and tissue in this robust systemic infection [58]. The high degree of overlap between feet on day 7 and 30 in both genes and pathways also argues that chronic arthritic disease represents a tailing off or extension of acute disease, rather than the activation of some fundamentally new inflammatory immunopathology [44]. This notion is further supported by the observation that only two of the genes, that were shared between days 2, 7 and 30 in feet, showed significantly higher fold change on day 30 than day 7. These were *Tnip3* and *Clec4d* (S1 Table), which are genes associated with inflammation resolution [114,115].

Interferome analysis of up-regulated genes provided a quantitative assessment of the dominance of the type I IFN response after CHIKV infection, with ≈50% of genes identified as type I IRGs at all time points and tissues tested except day 7 lymph nodes. This dominance of type I IRGs was retained (in feet) on day 7 and 30, despite the loss of type I IFN gene induction. The loss of type I IFN gene induction also occurred despite the persistence of viral RNA. Type I IFN-independent induction of type I IRGs (although well described [63,78,79]) thus entirely and seamlessly takes over after the brief period of type I IFN-dependent induction of IRGs. A number of transcription factors potentially responsible were identified; some were perhaps expected (e.g. IRF7, IRF3 [78,79,116]), whereas others (e.g. IRF1, IRF2, IRF5, IRF8) have not been extensively studied in alphavirus infections.

Therapeutic targeting of IFNγ would appear to have limited utility for treating CHIKV arthropathy, as CHIKV infection in IFNγ^-/-^ mice produces a relatively mild phenotype (reduced edema), despite abundant type II IRG induction and a robust IFNγ signature. The discrepancy may, at least in part, be explained by the transcription factor analysis, which suggested that genes induced via Stat:Stat1 can also be induced via other transcription factors (with several interferon response factors implicated). One might also speculate that the large volume of data sets on type II IRG induction may result in some pro-IFNγ bias in bioinformatics programs. Perhaps of note (given the similarities in the expression profiles of CHIKV and rheumatoid arthritis [27]), a phase II study of the anti-IFNγ agent, fontolizumab, in rheumatoid arthritis patients failed to show efficacy (ClinicalTrials.gov Identifier: NCT00281294).

Herein we describe the first phenotype of the recently generated GzmK^-/-^ mouse, which showed no evidence of anti-viral or cytolytic functions in lymphocytic choriomeningitis virus or ectromelia virus infections (manuscript in preparation). The reduction in foot swelling in CHIKV-infected GzmK^-/*-*^ mice reported here is consistent with previous suggestions of a non-cytolytic, pro-inflammatory role for granzyme K [117–119]. Granzyme A and granzyme K are related tryptic proteases that have arisen by gene duplication [120], and have overlapping substrate specificities [121].

Herein we illustrate for the first time that granzyme A is a key proinflammatory mediator during CHIKV arthritis, and (to our knowledge) are the first to show (in any setting) that inactivating granzyme A may have therapeutic benefit. The observation is consistent with the view that granzyme A’s proinflammatory role is associated with its proteolytic activity [92,96,103,104]. Our evidence argues against granzyme A having a significant role in suppressing CHIKV replication or in promoting viral clearance. Instead, our data is consistent with a number of studies in various systems that granzyme A promotes inflammation in both mice and humans [94,96,97,102–104,108,110,122–124]. Although a role for granzyme A in driving IL-1β-mediated inflammation in macrophages was recently reported [97], treatment with anakinra (a licensed IL-1 receptor antagonist) [125] provided only marginal amelioration of foot swelling in the CHIKV mouse model (manuscript in preparation).

The reduced arthritic disease in CHIKV-infected GzmA^-/-^ mice was associated with the reduction of NK cell and T cell infiltrates, with NK cells and CD4 T cells previously shown to promote arthritis in this model [27,30,65]. NK cells and CD4 T cells are also well described in human alphaviral arthritides [126,127], and both NK cells and cytotoxic CD4 T cells express and secrete granzyme A (as well as granzyme K) [128,129]. Although cytotoxic CD4 T cells are prominent in dengue infections [130], they have yet to be formally demonstrated in alphavirus infections. However, *CRTAM* was recently identified as critical for differentiation of cytotoxic CD4 T cells at sites of inflammation [93], and *CRTAM* was up-regulated in feet (but not lymph nodes) on days 2 and 7 with a fold change of 3.3 and 3.8, respectively (S1 Table). How loss of granzyme A expression by NK cells and T cells might lead to reduced numbers of these cells in arthritic lesions remains unclear. A role for granzyme A in type IV collagen degradation and lymphocyte migration has been reported [131], although migration through matrigel was not impaired in GzmA^-/-^ lymphocytes [132]. Perhaps of note, both granzyme A and K activate the protease activated receptor 1 (PAR1) [118,133], with PAR1 previously shown to be involved in inflammation [134,135], chemotaxis [136,137], and NK and T cell recruitment [138,139]. Granzyme A/K and thrombin (also known to activate PAR1) are trypsin-like proteases that cleave behind positively charged amino acids, with canonical proinflammatory signaling by PAR1 induced by cleavage at arginine 41 (Arg41) [140]. Granzyme B specifically cleaves behind aspartic acid residues; cleavage of PAR1 at Arg41 by granzyme B would thus be unlikely, perhaps explaining the lack of a CHIKV arthritis phenotype in GzmB^-/-^ mice.

Granzyme A (rather than IFNγ) from differentiated NK cells [126,141] and CD4 T cells [27,65] thus appears to be an important driver of CHIKV arthritis, with granzyme A dispensable for control of CHIKV infection [25]. We also show that granzyme A is elevated in CHIKV-infected NHPs and in CHIKV patients, with circulating granzyme A levels in NHPs peaking day 4–8 post infection, which coincides with the peak of circulating IFNγ levels [33]. Circulating granzyme A levels have previously been shown to be elevated in patients suffering from infections with dengue [105], EBV, HIV-1 [108], primary CMV [109], malaria [142], and bacteria [106,143], and also in rheumatoid arthritis patients [110]. Taken together these observations argue that granzyme A represents a potential target for anti-inflammatory interventions not only in alphaviral arthritides, but perhaps also in other inflammatory diseases.

## Methods

### Ethics statements

All mouse work was conducted in accordance with the “Australian code for the care and use of animals for scientific purposes” as defined by the National Health and Medical Research Council of Australia. Mouse work was approved by the QIMR Berghofer Medical Research Institute animal ethics committee (P1060 A705603M) and was conducted in biosafety level-3 facility at the QIMR Berghofer. Mice were euthanized using carbon dioxide.

NHP plasma samples were available from previous CHIKV studies [33,144]; a full ethics statement is provided in [144]. No additional NHPs were used for this study.

Human CHIKV serum samples were provided by the Centre for Infectious Diseases and Microbiology Laboratory Services (CIDMLS), Westmead Hospital (Sydney, Australia). Samples had been obtained from symptomatic patients who had returned to Australia from overseas and were collected for diagnostic purposes. All samples were IgM positive for CHIKV. Serum samples from healthy individuals were provided by the Australian Red Cross. Written and oral informed patient consent was obtained from all patients. No new human samples were collected as part of this study. Serum samples were deidentified before being provided for the research project and no patient data was provided or accessed. The study was approved by Griffith University Human Research Ethics Committee (BDD/01/12/HREC).

### Mice and CHIKV infection

C57BL/6J mice (6–8 weeks) were purchased from Animal Resources Center (Canning Vale, WA, Australia). Interferon-γ deficient mice (IFNγ^-/-^) mice (JR3288 B6.129S7-Ifng/J) were obtained from the Jackson Laboratory. Granzyme A deficient (GzmA^-/-^) and granzyme B deficient (GzmB^-/-^) mice were generated as described [145] and were backcrossed onto C57BL/6J mice a total of 12 times and were provided by the Peter MacCallum Cancer Centre, Melbourne, Victoria, Australia [146]. GzmK^-/-^ mice on a C57BL/6J background were provided by Prof Phillip Bird (manuscript submitted). Female mice were inoculated with 10^2^ or 10^4^ CCID_50_ of the Reunion Island isolate (LR2006-OPY1) in 40 μl of medium (RPMI1640 supplemented with 2% fetal calf serum), s.c. into both hind feet as described previously [13,28]. The virus (GenBank KT449801) was grown in C6/36 cells [13]. Serum viraemia was determined as described [9,13]. Foot swelling was measured using digital calipers and is presented as a group average of the percentage increase in foot height times width for each foot compared with the same foot on day 0 [13].

### RNA isolation for RNA-Seq analysis

C57BL/6 mice were infected with 10^4^ CCID_50_ CHIKV as described above and whole feet (cut above the ankle) and inguinal lymph nodes harvested on days 2, 7 and (for feet) 30 post infection. Mock infected mice were injected s.c. in the feet (i) with medium (and harvested 2 days later) or (ii) with heat inactivated (60°C, 30 mins) viral inocula (and harvested on day 30). Tissues were placed in RNAlater (Life Technologies) overnight at 4°C and then homogenized in TRIzol (Invitrogen) using 4 x 2.8 mm ceramic beads (MO BIO Inc., Carlsbad, USA) and a Precellys24 Tissue Homogeniser (Bertin Technologies, Montigny-le-Bretonneux, France) (3 x 30 s, 6000 rpm on ice). Homogenates were centrifuged (12,000 g x 10 min) and RNA extracted from the supernatants as per manufacturer’s instructions. RNA concentration and purity was determined by Nanodrop ND 1000 (NanoDrop Technologies Inc.). Eight RNA pools were generated in triplicate with each of the 24 samples containing equal amounts of RNA from four different mice; (i) feet day 2, (ii) feet day 7, (iii) mock feet day 2, (iv) feet day 30, (v) mock feet day 30, (vi) lymph node day 2, (vii) lymph node day 7 and (viii) mock lymph node day 2. The 24 samples were DNase treated using RNAse-Free DNAse Set (Qiagen), purified using an RNeasy MinElute Kit, and sent to the Australian Genome Research Facility (Melbourne, Australia).

### RNA-Seq analysis

Library preparation and sequencing were conducted by the Australian Genome Research Facility (Melbourne, Australia). cDNA libraries were prepared using a TruSeq RNA Sample Prep Kit (v2) (Illumina Inc. San Diego, USA), which includes isolation of poly-adenylated RNA using oligo-dt beads. cDNA libraries were mixed and sequenced from both ends (100 bp) using Illumina HiSeq 2000 Sequencer (Illumina Inc.). To obtain a high sequencing depth (total ≈55,000,000 paired end reads per sample) each library was sequenced three times using independent lanes. The CASAVA v1.8.2 pipeline was used to separate the bar-coded sequences and extract 100 base pair, paired end reads into FASTQ files.

### Differentially expressed genes

Bowtie v2.0.2 and Tophat v2.0.6 [147,148] were used to align paired end read sequences to the UCSC *mus musculus* full genome build (mm10, Dec. 2011) to generate bam files (default parameters). The Cufflinks suite v2.1.1 (default parameters) [148,149] was then used to assemble transcripts (MapQ > 20) and calculate relative abundance and generate differentially expressed gene (DEG) lists. Differential gene expression for day 2 and 7 post CHIKV infection was determined relative to mock inoculated mice that had received medium 2 days previously, and differential gene expression for day 30 post CHIKV infection was determined relative to mice that had received heat inactivated viral inocula 30 days previously. For further analysis, DEGs were selected where (i) the q-value (false discovery rate adjusted p-value) was < 0.01, (ii) the fold change was > 2 relative to mock and (iii) FPKM was > 1 in the mock or the infected sample [74,75]. DEGs were analyzed by the Database for Annotation, Visualization and Integrated Discovery (DAVID) v6.7 [150], Ingenuity Pathway Analysis (IPA; Ingenuity Systems) and Interferome v2.01 [53].

### CHIKV genome alignment

Reads that did not map to the mouse genome where aligned to the CHIKV genome (LR2006-OPY1; GenBank KT449801) (excluding the polyA tail) using Bowtie v2.0.2. The frequency allele threshold was set to 5% with a mapQ>20. Reads alignments were visualized using the Integrative Genomics Viewer (IGV) version 2.3.34 [151]. Single nucleotide polymorphism analyses of the CHIKV sequences was undertaken using Geneious v. 7.1.5 [152] using minimum coverage of 20 reads per position and a minimum variant frequency of 0.5%.

### Transcription factor binding site analysis

Gene lists were analyzed by the recently developed software, CiiiDER (Gearing et al., in prep), which predicts key transcription factors regulating co-expressed genes. Using motifs released by TRANSFAC (2011), the software used a Java-based implementation of the Match algorithm [153] to identify putative tissue factor binding sites in sets of up-regulated gene, as well as in sets of background genes (for each time point), whose expression was not significantly changed by viral infection. A Fisher's exact test was used to identify sites significantly over-represented (enriched) in the up-regulated genes compared to the background genes and to provide p values as described [154].

### Histology and immunohistochemistry

Histology, immunohistochemistry and quantitation was performed as described previously [13,28,46]. Briefly, feet were fixed in paraformadehyde, decalcified and embedded in paraffin, and sections stained with hematoxylin and eosin (H&E). For immunohistochemistry, sections were stained with anti-NKp46 (rabbit polyclonal; Biorybt, Berkeley, CA) or anti-CD3 (A0452; Dako, North Sydney, Australia), with detection using MACH 2 (Biocare, Concord, CA) and Nova Red. F4/80 staining was undertaken as described [28]. Sections were scanned using Aperio AT Turbo (Aperio, Vista, CA) and analyzed using Aperio ImageScope software (v10) and the Positive Pixel Count v9 algorithm.

### Granzyme A inhibitor, recombinant Serpinb6b

His-tagged recombinant Serpinb6b was produced at the Monash University Protein Production Unit using *Pichia pastoris* and purified using a nickel column followed by HiTrap Q column anion exchange chromatography (GE Healthcare Life Sciences) [107,155]. As a negative control the recombinant Serpinb6b was digested with tissue culture grade trypsin (Sigma) (1:1 molar ratio) for 30 mins at 37°C prior to injection. Serpinb6b (0.6 mg/ml) was diluted in RPMI 1640 and injected i.v. daily, 10 μg in 100 μl.

### Granzyme A protein levels in primates

Plasma samples were available from previous experiments in which *Macaca fascicularis* NHPs had been infected with a range of doses of CHIKV as described [33,144]. Granzyme levels were determined using Monkey Granzyme A and K ELISA Kits (MyBioSource, San Diego, CA) according to manufacturer’s instructions. Viral loads were measured by quantative RT PCR as described [33].

Human serum samples were tested for granzyme A using the Human Granzyme A Flex Set (BD Cytometric Bead Array) and the LSRFortessa Cell Analyser (BD Biosciences, San Diego, CA, USA) according to manufacturer’s protocols.

### Statistics

Statistical was performed using IBM SPSS Statistics (version19). The t test was used if the difference in the variances was <4, skewness was >-2, and kurtosis was <2; where the data was nonparametric and difference in variances was <4, the Mann Whitney U test was used, if >4 the Kolmogorov-Smirnov test was used [13]. A 2 way ANOVA was used for some Aperio data and included a term for section. For NHP data paired t tests, Pearson and Spearman correlations were also used. The Kruskal-Wallis test was used for human serum granzyme A levels.

## Supporting information

S1 Fig Quality control analyses and read alignment data. (A) A raw data quality analysis for paired end reads; total number of nucleotides sequenced was 675,021,384 paired reads x 100 b.p. per read x 2 paired end reads (top graph for forward reads, the bottom graph reverse reads). The analysis was undertaken using the FastQC program (http://www.bioinformatics.babraham.ac.uk/projects/fastqc/) (v 0.11.4). The vast majority of reads were of high quality (green zone). The figure is representative of forward and reverse reads for all 3 sequencing runs. No reads required trimming prior to analysis. (B) RLE plot illustrating normalization of all data sets. The box plot was generated by the function plotRLE in R package “EDASeq” [156], which produces a Relative Log Expression (RLE) plot of the counts illustrating the differences between the distributions of read counts across samples. (C) PCA plot. The PCA plot shows clustering of biological triplicates for foot and control samples. Day 2/7 and Day 30 samples were are derived from separate experiments and were sequenced on separate sequencing runs; Day 2 mock represents injection with medium day 0 and harvesting day 2, and Day 30 mock represents injection of heat inactivated virus in medium on day 0 and harvesting on day 30. The PCA plot was generated by the function plotPCA in R package “EDASeq”. (D) Read alignment data (MapQ > 20) to the mouse genome, mm10 (UCSC Mus musculus full genome build; Dec. 2011), and the CHIKV genome (LR2006-OPY1; GenBank KT449801) (excluding the polyA tail). Twenty four libraries were sequenced, representing 8 samples each with 3 biological replicates (each representing pooled samples from 4 mice). Read alignment data for the 2 experiments is shown. ^1^Both paired end read mates mapped to the mm10 genome. ^2^Neither read mate mapped to the mm10 genome.
(PDF)

S2 Fig IPA canonical pathway analysis. IPA canonical pathway analysis of up-regulated genes (only pathways where ≥4 DEGs are present on at least one time point are shown). The p values are shown as−log_10_ p values. Where−log_10_ p <1.3 (p>0.05) the pathway is indicted with yellow and grey in the heat map.
(PDF)

S3 Fig The CHIKV genome. (A) Examples of alignments of RNA-Seq reads from 2 lymph node samples mapped to the CHIKV genome (mapQ ≥ 20) viewed using Integrated Genomics Viewer. (B) Mutation analysis showing three sets of graphs; % change, Coverage, and % change vs coverage. % change; for each nucleotide position in the CHIKV genome and for each of the 3 biological replicates (represented in green, purple and blue), the percentage of reads showing a different nucleotide from the parental sequence was calculated. Only nucleotide positions which had at least 20 reads covering that position were included. MapQ >20 was used. Percentage values >0.5% are shown. “Coverage” shows the read coverage for each replicate and represents the number of reads obtained for each nucleotide position in the CHIKV genome. Data for day 7 lymph node is not shown as read coverage was too low. The “% change vs coverage” represents the % of reads (for a given position in the genome) showing a nucleotide change inversely correlated with read coverage. (Note x axis label for day 2 lymph node is not x1000). (C) Graph showing the percentage of changes that were non- synonymous for CHIKV sequences from each tissue and time point. For day 7 lymph nodes there was insufficient sequence data for amino acid coding regions.
(PDF)

S4 Fig Bar charts of the data shown in Fig 1F. Error bars show variance between the three pooled biological replicates. Note mock infection samples for day 2/7 are distinct from mock infection samples for day 30.
(PDF)

S5 Fig Transcription factor usage for up-regulated genes in feet. (A) Ingenuity upstream regulator analysis−log_10_ p values for the data shown in Fig 3D. (B) Using the same DEG sets as in Fig 3D, a new program (CiiiDER, Gearing et al, in prep) was used to determine what putative transcription factor sites (motifs provided by TRANSFAC) were predicted to be significantly enriched in promoters of up-regulated genes when compared with promoters in control genes, whose mRNA abundance was not significantly altered by CHIKV infection. (TRANSFAC has no motifs for IRF5).
(PDF)

S6 Fig IFNγ^-/-^ mice. (A) Viraemia in IFNγ^-/-^ mice infected with 2 different doses of the Reunion Island isolate of CHIKV. At the higher dose (left) no significant differences were observed (n = 5 KO and 10 C57BL/6 mice) [27]. At a lower dose (right) the viraemia was significantly higher (red arrow) in IFNγ^-/-^ mice on day 5 post infection (n = 6 mice per group, statistics by Mann Whitney U test). (B) Foot swelling in IFNγ^-/-^ mice with low dose CHIKV inoculums was significantly increased (red arrow). Statistics by t test; * p<0.004, # p = 0.01, (n = 6 mice per group). (C) Overt subcutaneous edema in foot sections measured using Aperio pixel count (3 sections per foot, n = 6 feet from 6 mice, statistics by Kolmogorov-Smirnov test). (D) H & E staining showing overt subcutaneous edema in wild-type (black oval), but not IFNγ^-/-^ mice.
(PDF)

S7 Fig Putative transcription factor site analysis of up-regulated type II IRGs in feet. (A) Formulas for calculating x and y values plotted in Fig 3E. The x axis provides a measure of the proportion of the genes with the putative transcription factor site in their promoter. The y axis provides a measure of the over or under-representation of genes with the putative transcription factor in each IRG gene set. (B) The data plotted in Fig 3E in table form, with transcription factor motif identifiers (TRANFAC) and input/output data provided.
(PDF)

S8 Fig GzmA^-/-^ mice; repeat experiment, persistent CHIKV RNA and antibody responses. (A) Independent repeat experiment comparing viremia (left) and foot swelling (right) in GzmA^-/-^ mice and C57BL/6 control mice. There were no significant differences in the viraemias. The foot swelling was significantly lower (red arrow) in GzmA^-/-^ mice vs C57BL/6 control mice on days 1–10 (n = 5/6 mice per group; Kolmogorov-Smirnov and Mann Whitney U tests, * p = 0.023, ** p<0.003). (B) Quantitative RT PCR of CHIKV RNA from mouse feet day 30 post infection undertaken as described [13]; n = 6 feet from 6 mice per group. (C) Antibody responses in serum day 30 post infection in GzmA^-/-^ mice and C57BL/6 mice. Results from 2 independent experiments are shown (n = 5/6 mice per group).
(PDF)

S9 Fig Plasma granzyme A and K levels in CHIKV-infected NHPs. (A) As for Fig 6E but including data from all 11 animals. (B) Mean granzyme A levels using data from all NHPs plotted over time; includes data for an additional NHP for whom day -1 data was not available (n = 12). Differences between day -1 and day 7 were significantly different (t test). (C) As for B for plasma granzyme K levels. (D) Viral loads as determined by qRT PCR for the 9 NHPs shown in Fig 6D. (E) Dot plot of all plasma samples for which both viral load and granzyme A levels were available; each data point shows the granzyme A level and the viral load in one sample. (F) Correlation between peak viral loads (log) and peak granzyme A levels. Statistics by Pearson correlation.
(PDF)

S1 Table Differentially expressed gene (DEG) lists. DEG lists for feet (Ft) (days 2, 7, and 30) and lymph node (LN) (days 2 and 7) where fold change (FC) >2 (i.e. log_2_ FC >1) and q<0.01 are provided. Subsequent tabs contain lists of up and down regulated DEGs, with an additional filter whereby only DEGs are listed where FPKM>1 for at least one of the two time points in the pair wise comparisons. Where FC is infinite, a nominal log_2_ FC value of 21 has been entered. For day 7 LN, immunoglobulin genes have been highlighted in yellow.
(XLSX)

S2 Table Concordance of up-regulated genes identified by RNA-Seq in the current study of CHIKV infected mice and mRNA and protein expression studies in CHIKV infected mice and monkeys. (DOCX)

S3 Table Gene lists from the bioinformatics analyses. The gene lists for Fig 1A (the 247 shared genes), Fig 1B, 1D and 1F are provided.
(XLSX)
