# Supplementary material for: RNA-Seq analysis of chikungunya virus infection and identification of granzyme A as a major promoter of arthritic inflammation
Source: PLoS Pathog. 2017 Feb 16;13(2):e1006155. doi: 10.1371/journal.ppat.1006155 (PMC5312928; doi:10.1371/journal.ppat.1006155)
Supplement: S1 Fig — (A) A raw data quality analysis for paired end reads; total number of nucleotides sequenced was 675,021,384 paired reads x 100 b.p. per read x 2 paired end reads (top graph for forward reads, the bottom graph reverse reads). The analysis was undertaken using the FastQC program (http://www.bioinformatics.babraham.ac.uk/projects/fastqc/) (v 0.11.4). The vast majority of reads were of high quality (green zone). The figure is representative of forward and reverse reads for all 3 sequencing runs. No reads required trimming prior to analysis. (B) RLE plot illustrating normalization of all data sets. The box plot was generated by the function plotRLE in R package “EDASeq” [156], which produces a Relative Log Expression (RLE) plot of the counts illustrating the differences between the distributions of read counts across samples. (C) PCA plot. The PCA plot shows clustering of biological triplicates for foot and control samples. Day 2/7 and Day 30 samples were are derived from separate experiments and were sequenced on separate sequencing runs; Day 2 mock represents injection with medium day 0 and harvesting day 2, and Day 30 mock represents injection of heat inactivated virus in medium on day 0 and harvesting on day 30. The PCA plot was generated by the function plotPCA in R package “EDASeq”. (D) Read alignment data (MapQ > 20) to the mouse genome, mm10 (UCSC Mus musculus full genome build; Dec. 2011), and the CHIKV genome (LR2006-OPY1; GenBank KT449801) (excluding the polyA tail). Twenty four libraries were sequenced, representing 8 samples each with 3 biological replicates (each representing pooled samples from 4 mice). Read alignment data for the 2 experiments is shown. 1Both paired end read mates mapped to the mm10 genome. 2Neither read mate mapped to the mm10 genome. (PDF) [file ppat.1006155.s001.pdf]

S1 Fig.

A

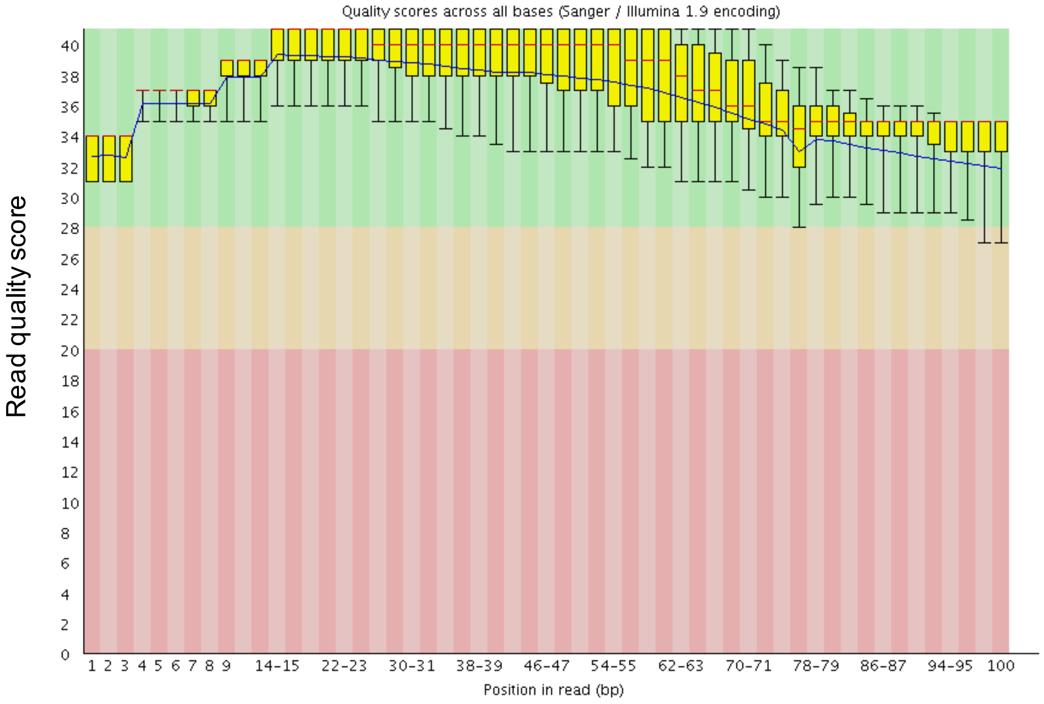

Quality scores across all bases (Sanger / Illumina 1.9 encoding)
Read quality score
0 2 4 6 8 10 12 14 16 18 20 22 24 26 28 30 32 34 36 38 40
1 2 3 4 5 6 7 8 9 14-15 22-23 30-31 38-39 46-47 54-55 62-63 70-71 78-79 86-87 94-95 100
Position in read (bp)

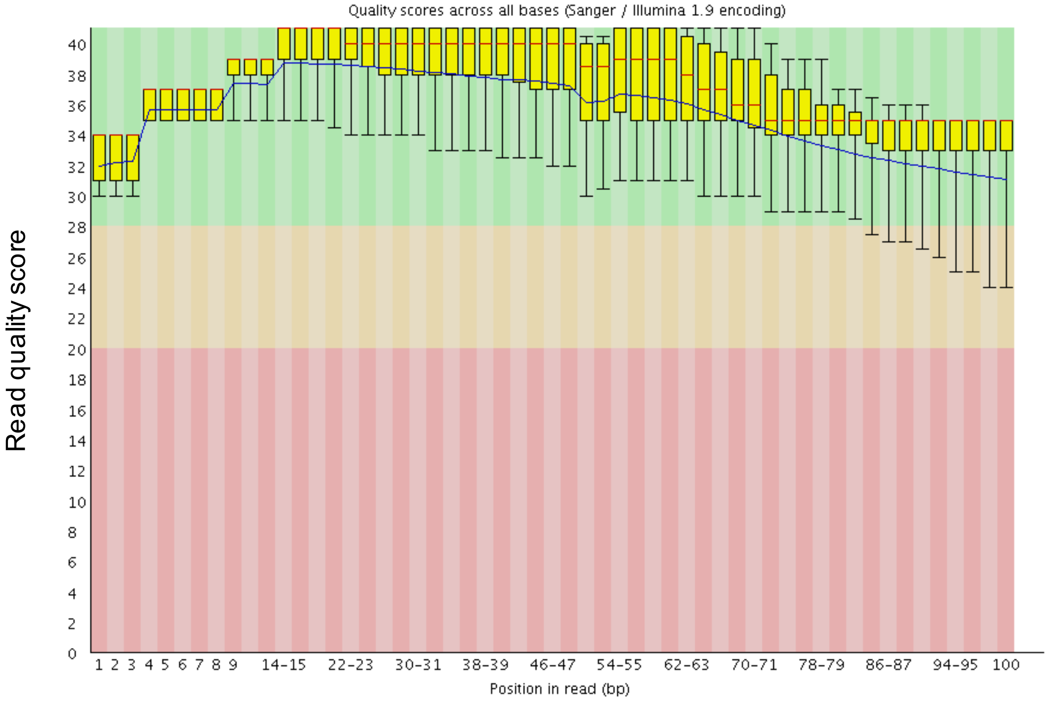

Quality scores across all bases (Sanger / Illumina 1.9 encoding)
Read quality score
0 2 4 6 8 10 12 14 16 18 20 22 24 26 28 30 32 34 36 38 40
1 2 3 4 5 6 7 8 9 14-15 22-23 30-31 38-39 46-47 54-55 62-63 70-71 78-79 86-87 94-95 100
Position in read (bp)

S1 Fig.

B

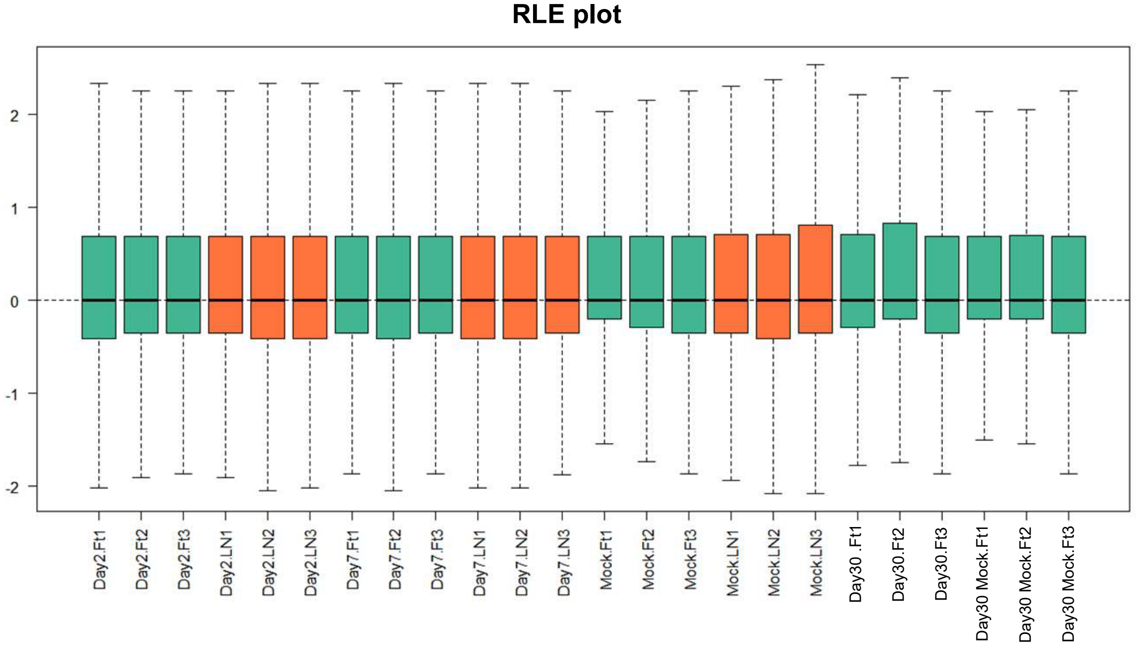
RLE plot
2
1
0
-1
-2
Day2.Ft1
Day2.Ft2
Day2.Ft3
Day2.LN1
Day2.LN2
Day2.LN3
Day7.Ft1
Day7.Ft2
Day7.Ft3
Day7.LN1
Day7.LN2
Day7.LN3
Mock.Ft1
Mock.Ft2
Mock.Ft3
Mock.LN1
Mock.LN2
Mock.LN3
Day30 .Ft1
Day30.Ft2
Day30.Ft3
Day30 Mock.Ft1
Day30 Mock.Ft2
Day30 Mock.Ft3

C

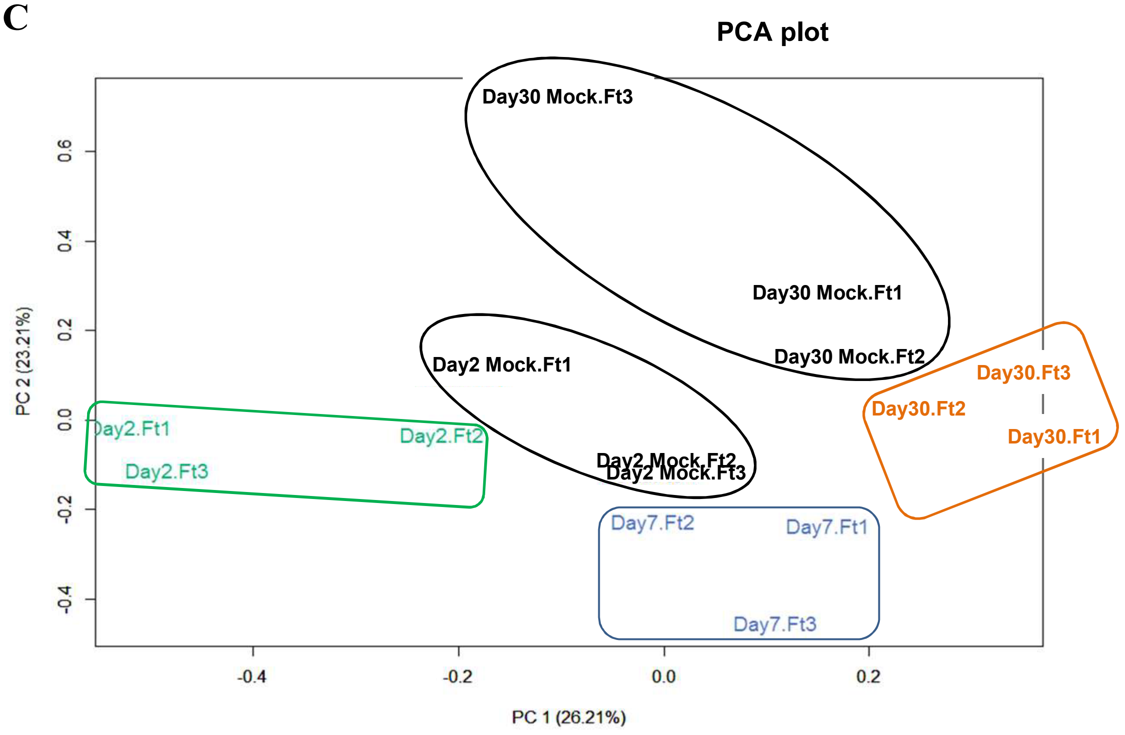
PCA plot
Day30 Mock.Ft3
Day30 Mock.Ft1
Day30 Mock.Ft2
Day2 Mock.Ft1
Day2 Mock.Ft2
Day2 Mock.Ft3
Day30.Ft3
Day30.Ft2
Day30.Ft1
Day2.Ft1
Day2.Ft2
Day2.Ft3
Day7.Ft2
Day7.Ft1
Day7.Ft3
0.6
0.4
0.2
0.0
-0.2
-0.4
PC 2 (23.21%)
-0.4
-0.2
0.0
0.2
PC 1 (26.21%)

S1 Fig.

D

Sample	Total reads; mean of 3 biological replicates. (SD)	Reads aligned to mm10; mean of 3 biological replicates (% ± SD)[1]	Reads aligned to CHIKV; mean of 3 biological replicates (% ± SD)[2]
Day 2 feet	55,349,451 (715,780)	46,620,761 (84.231 ± 0.612)	4,572,386 (8.260 ± 0.389)
Day 2 lymph node	52,331,910 (2,497,885)	47,039,395 (89.881 ± 0.293)	4,094 (0.008 ± 0.003)
Day 7 feet	57,669,081 (1,359,085)	52,598,842 (91.208 ± 0.078)	77,219 (0.134 ± 0.015)
Day 7 lymph node	53,248,035 (1,071,534)	47,588,454 (89.373 ± 0.146)	1,444 (0.003 ± 0.000)
Day 2 mock feet	51,011,141 (1,550,329)	46,188,873 (90.551 ± 0.612)	-
Day 2 mock lymph node	54,767,209 (1,789,228)	49,093,156 (89.638 ± 0.251)	-

Sample	Total reads; mean of 3 biological replicates (SD)	Reads aligned to mm10; mean of 3 biological replicates (% ± SD)[a]	Reads aligned to CHIKV; mean of 3 biological replicates (% ± SD)[b]
Day 30 feet	67,818,092 (5,656,088)	61,389,281 (90.515 ± 0.785)	2219 (0.105 ± 0.014)
Day 30 mock	57,819,338 (2,677,119)	49,553,702 (85.764 ± 7.759)	-
