# Supplementary figures and images for: RNA-Seq analysis of chikungunya virus infection and identification of granzyme A as a major promoter of arthritic inflammation
Source: PLoS Pathog. 2017 Feb 16;13(2):e1006155. doi: 10.1371/journal.ppat.1006155 (PMC5312928; doi:10.1371/journal.ppat.1006155)

## S2 Fig.

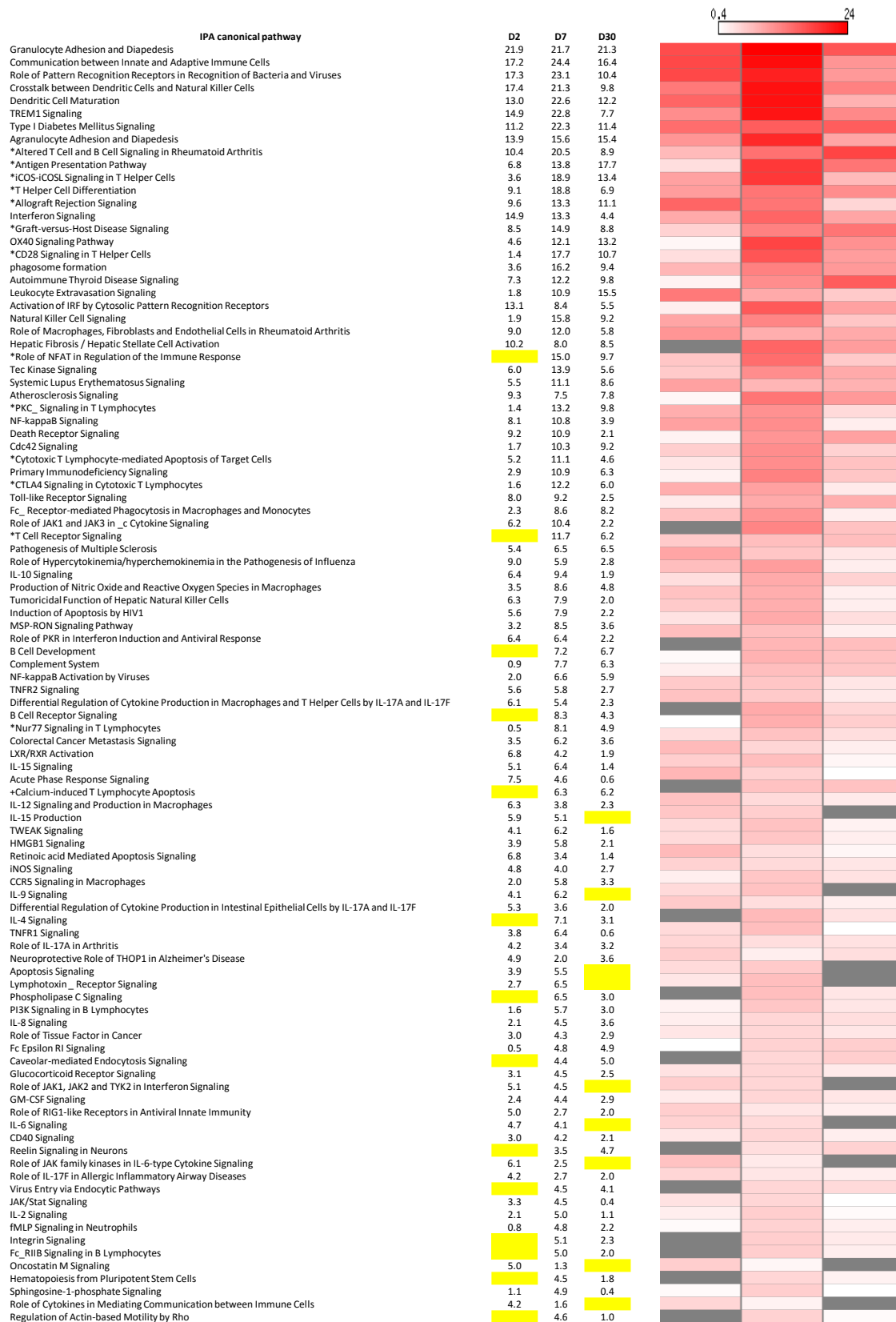

Supplement: S2 Fig — IPA canonical pathway analysis of up-regulated genes (only pathways where ≥4 DEGs are present on at least one time point are shown). The p values are shown as−log10 p values. Where−log10 p <1.3 (p>0.05) the pathway is indicted with yellow and grey in the heat map. (PDF) [file ppat.1006155.s002.pdf]

S3 Fig.

A

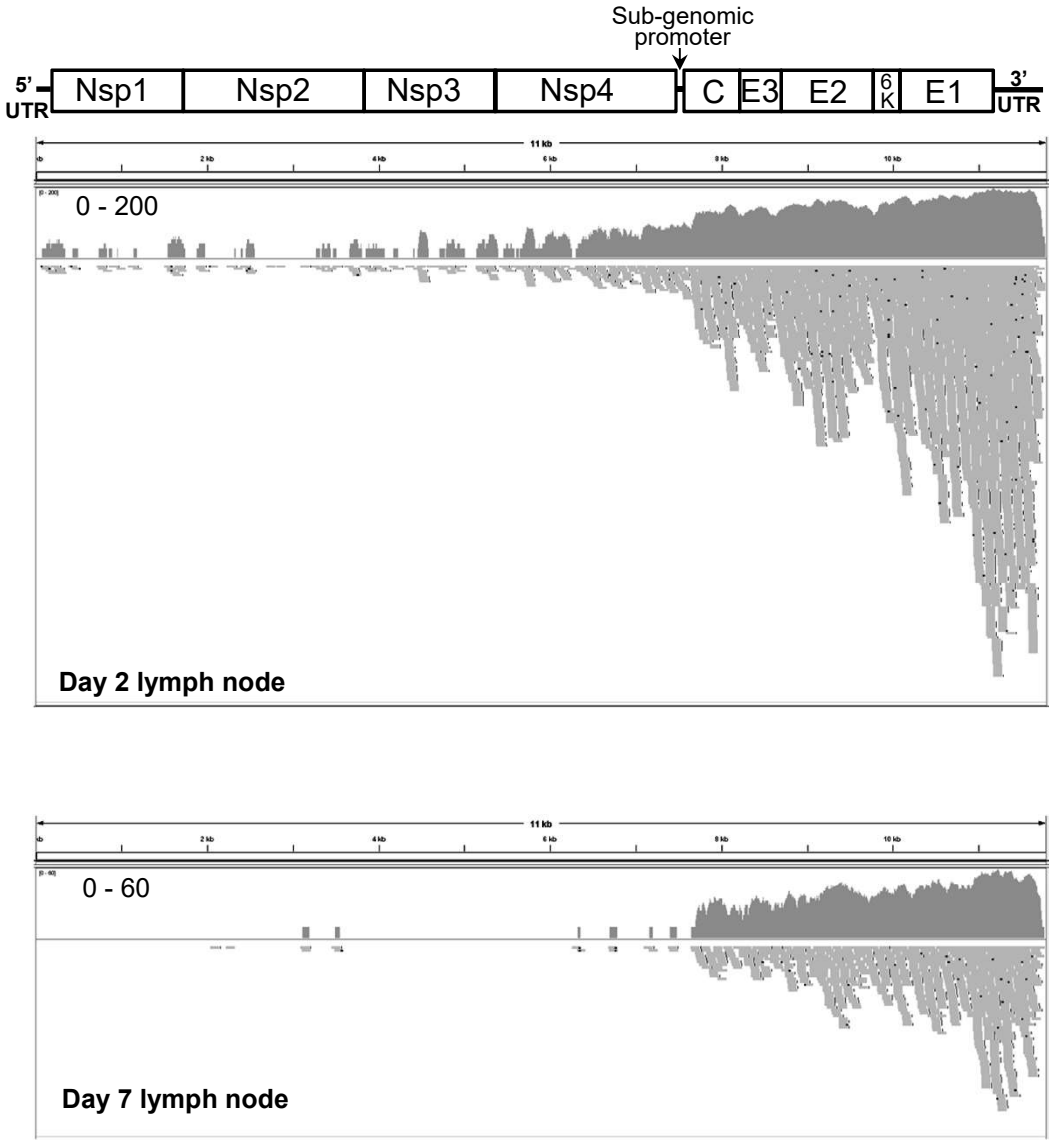

**S3 Fig.**

**B**

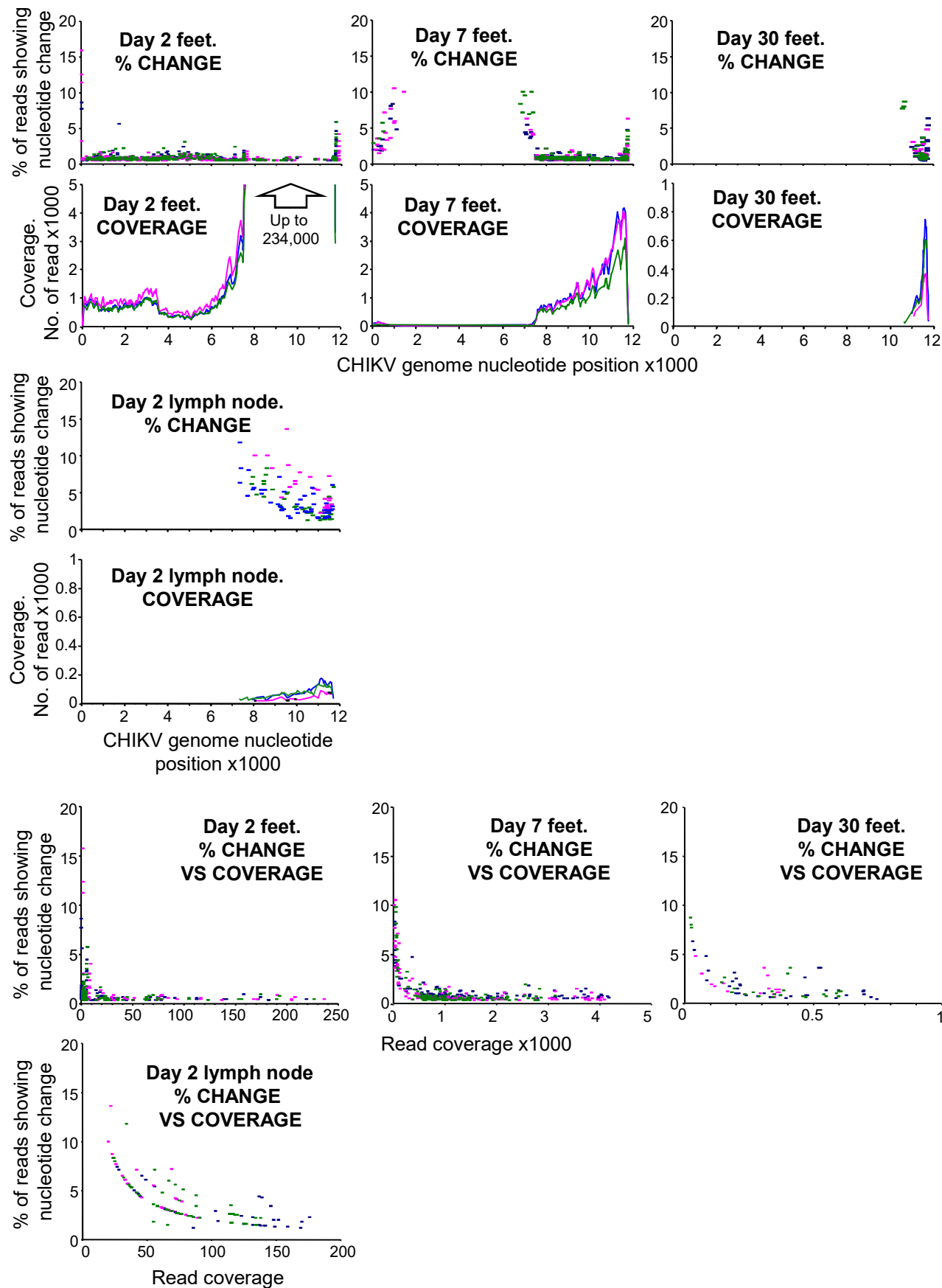

**S3 Fig.**

**C**

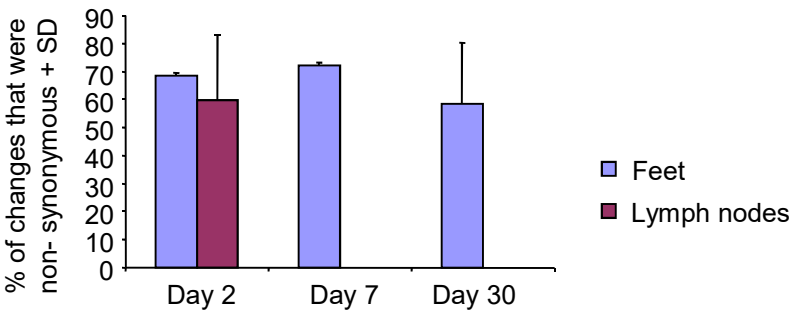

Supplement: S3 Fig — (A) Examples of alignments of RNA-Seq reads from 2 lymph node samples mapped to the CHIKV genome (mapQ ≥ 20) viewed using Integrated Genomics Viewer. (B) Mutation analysis showing three sets of graphs; % change, Coverage, and % change vs coverage. % change; for each nucleotide position in the CHIKV genome and for each of the 3 biological replicates (represented in green, purple and blue), the percentage of reads showing a different nucleotide from the parental sequence was calculated. Only nucleotide positions which had at least 20 reads covering that position were included. MapQ >20 was used. Percentage values >0.5% are shown. “Coverage” shows the read coverage for each replicate and represents the number of reads obtained for each nucleotide position in the CHIKV genome. Data for day 7 lymph node is not shown as read coverage was too low. The “% change vs coverage” represents the % of reads (for a given position in the genome) showing a nucleotide change inversely correlated with read coverage. (Note x axis label for day 2 lymph node is not x1000). (C) Graph showing the percentage of changes that were non- synonymous for CHIKV sequences from each tissue and time point. For day 7 lymph nodes there was insufficient sequence data for amino acid coding regions. (PDF) [file ppat.1006155.s003.pdf]

S4 Fig.

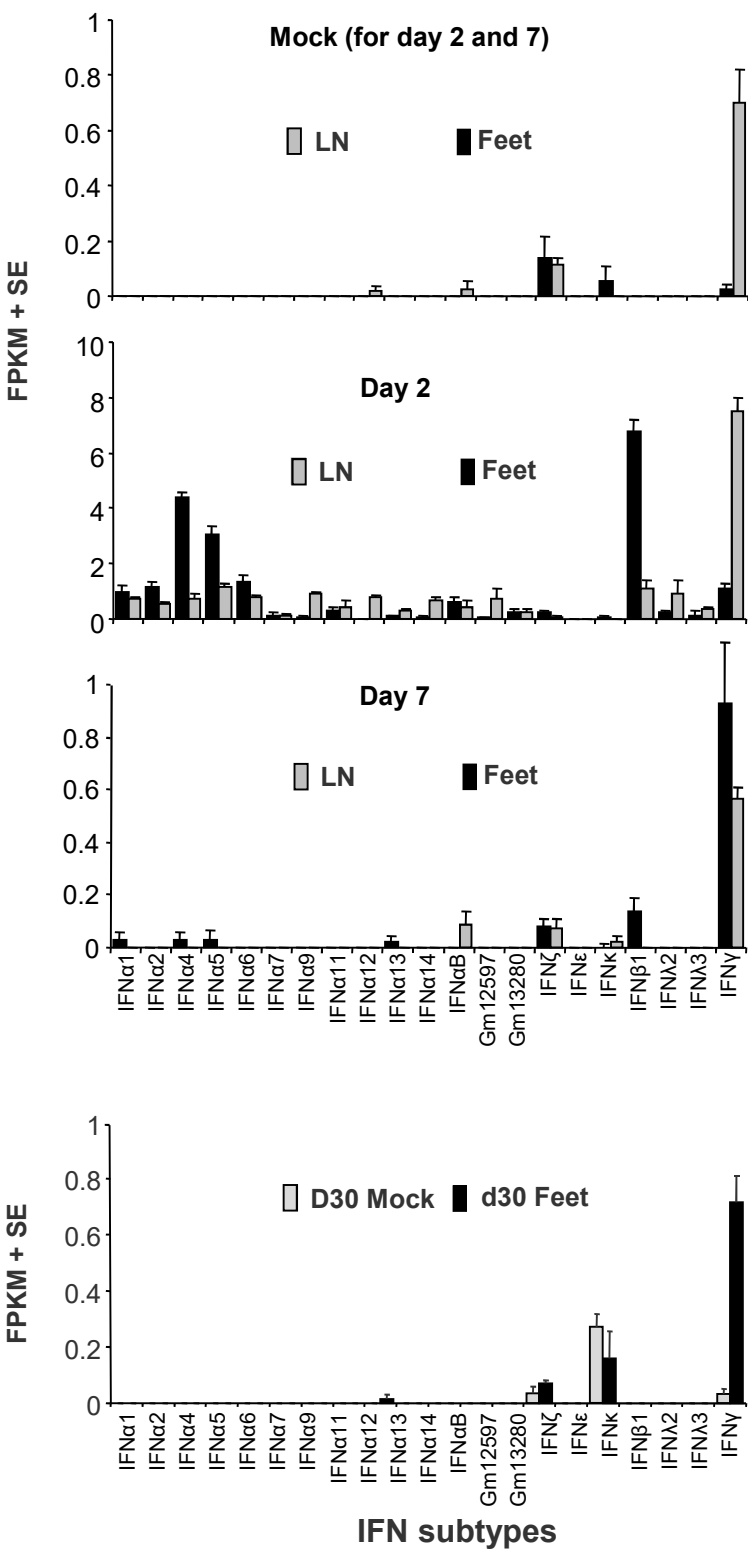

Supplement: S4 Fig — Error bars show variance between the three pooled biological replicates. Note mock infection samples for day 2/7 are distinct from mock infection samples for day 30. (PDF) [file ppat.1006155.s004.pdf]

**S6 Fig.**

**A**

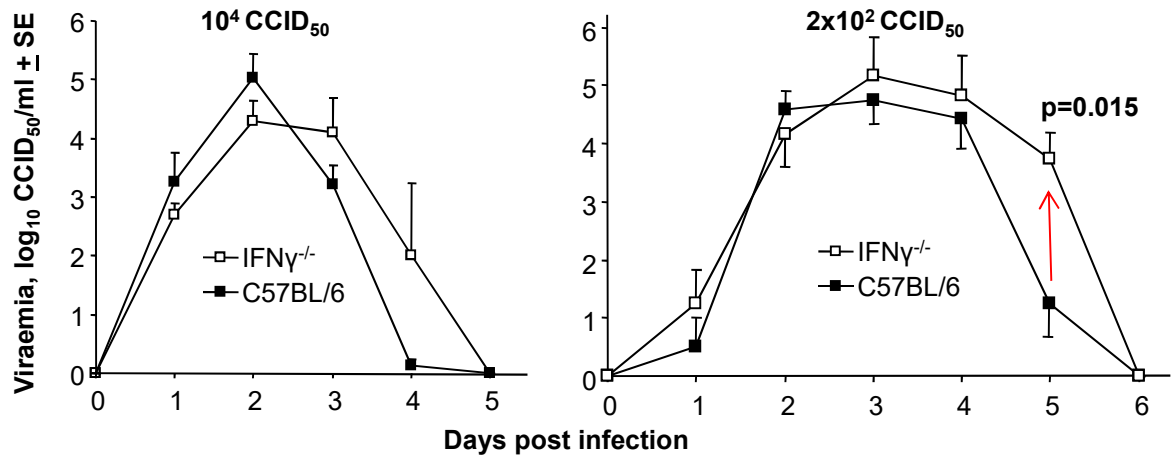

**B**

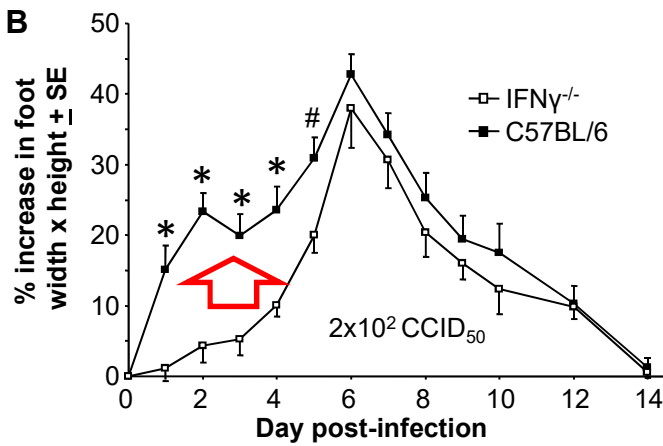

**C**

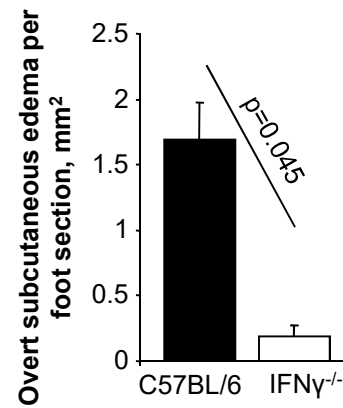

**D**

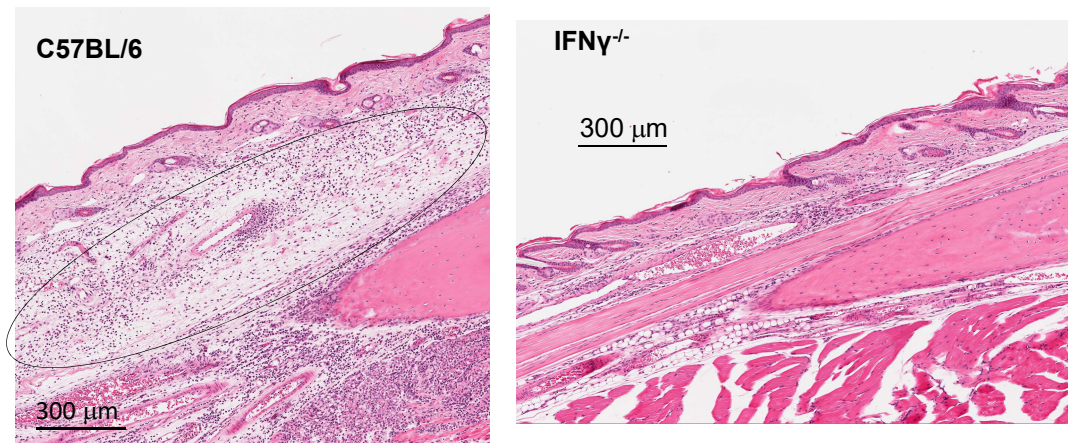

Supplement: S6 Fig — (A) Viraemia in IFNγ-/- mice infected with 2 different doses of the Reunion Island isolate of CHIKV. At the higher dose (left) no significant differences were observed (n = 5 KO and 10 C57BL/6 mice) [27]. At a lower dose (right) the viraemia was significantly higher (red arrow) in IFNγ-/- mice on day 5 post infection (n = 6 mice per group, statistics by Mann Whitney U test). (B) Foot swelling in IFNγ-/- mice with low dose CHIKV inoculums was significantly increased (red arrow). Statistics by t test; * p<0.004, # p = 0.01, (n = 6 mice per group). (C) Overt subcutaneous edema in foot sections measured using Aperio pixel count (3 sections per foot, n = 6 feet from 6 mice, statistics by Kolmogorov-Smirnov test). (D) H & E staining showing overt subcutaneous edema in wild-type (black oval), but not IFNγ-/- mice. (PDF) [file ppat.1006155.s006.pdf]

S8 Fig.

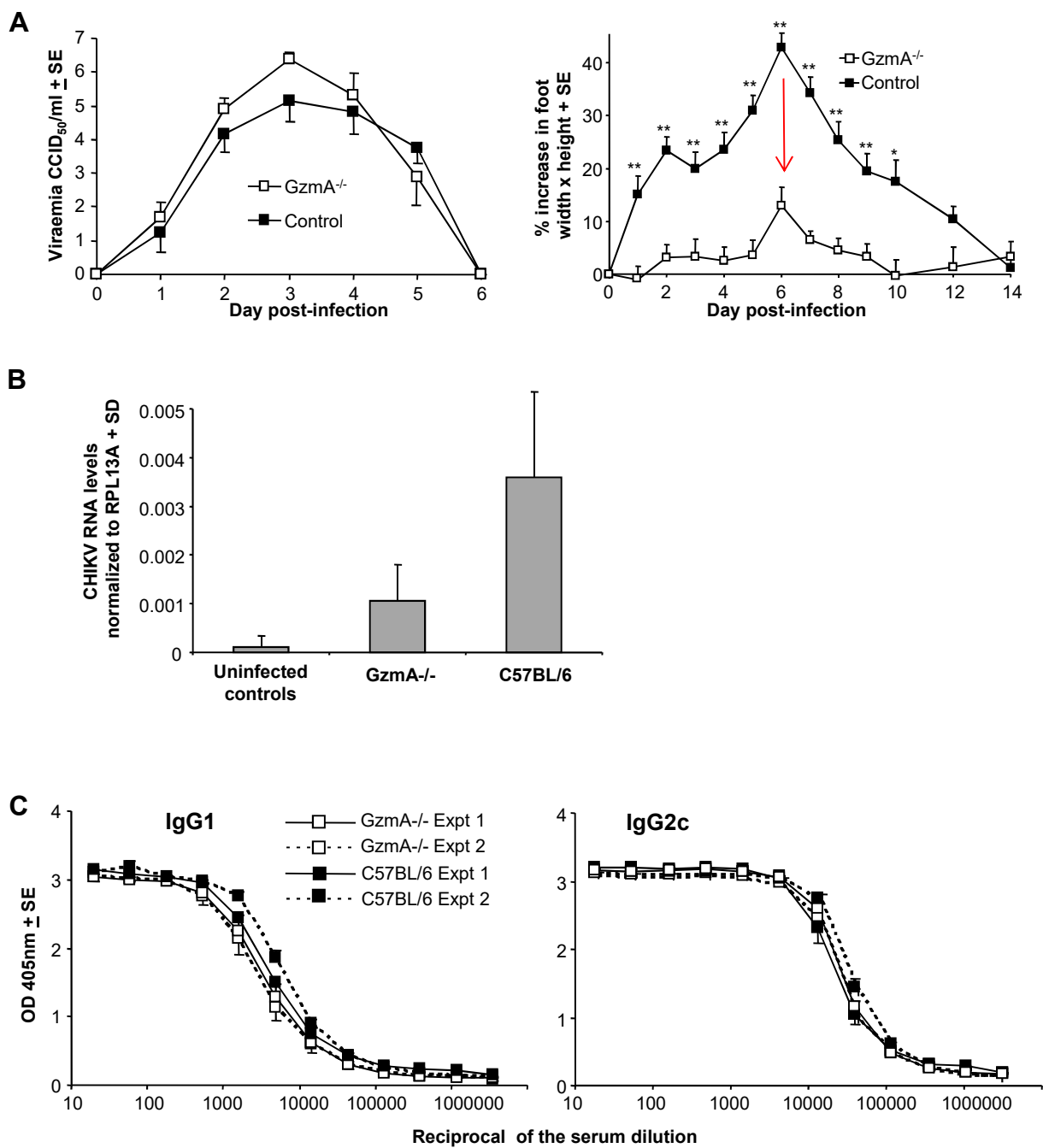

Supplement: S8 Fig — (A) Independent repeat experiment comparing viremia (left) and foot swelling (right) in GzmA-/- mice and C57BL/6 control mice. There were no significant differences in the viraemias. The foot swelling was significantly lower (red arrow) in GzmA-/- mice vs C57BL/6 control mice on days 1–10 (n = 5/6 mice per group; Kolmogorov-Smirnov and Mann Whitney U tests, * p = 0.023, ** p<0.003). (B) Quantitative RT PCR of CHIKV RNA from mouse feet day 30 post infection undertaken as described [13]; n = 6 feet from 6 mice per group. (C) Antibody responses in serum day 30 post infection in GzmA-/- mice and C57BL/6 mice. Results from 2 independent experiments are shown (n = 5/6 mice per group). (PDF) [file ppat.1006155.s008.pdf]

S9 Fig.

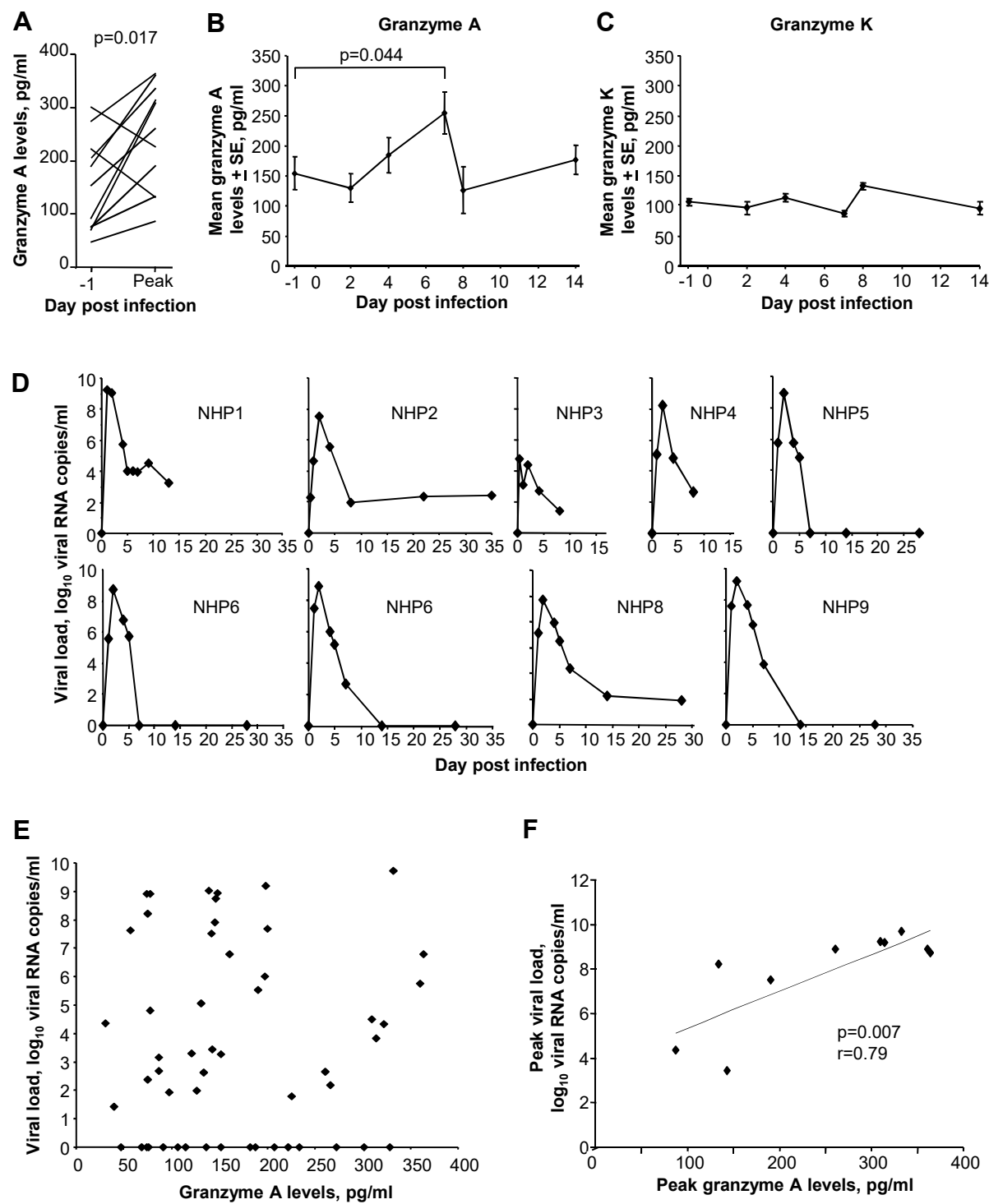

Supplement: S9 Fig — (A) As for Fig 6E but including data from all 11 animals. (B) Mean granzyme A levels using data from all NHPs plotted over time; includes data for an additional NHP for whom day -1 data was not available (n = 12). Differences between day -1 and day 7 were significantly different (t test). (C) As for B for plasma granzyme K levels. (D) Viral loads as determined by qRT PCR for the 9 NHPs shown in Fig 6D. (E) Dot plot of all plasma samples for which both viral load and granzyme A levels were available; each data point shows the granzyme A level and the viral load in one sample. (F) Correlation between peak viral loads (log) and peak granzyme A levels. Statistics by Pearson correlation. (PDF) [file ppat.1006155.s009.pdf]
