# Supplementary material for: RNA-Seq analysis of chikungunya virus infection and identification of granzyme A as a major promoter of arthritic inflammation
Source: PLoS Pathog. 2017 Feb 16;13(2):e1006155. doi: 10.1371/journal.ppat.1006155 (PMC5312928; doi:10.1371/journal.ppat.1006155)
Supplement: S5 Fig — (A) Ingenuity upstream regulator analysis−log10 p values for the data shown in Fig 3D. (B) Using the same DEG sets as in Fig 3D, a new program (CiiiDER, Gearing et al, in prep) was used to determine what putative transcription factor sites (motifs provided by TRANSFAC) were predicted to be significantly enriched in promoters of up-regulated genes when compared with promoters in control genes, whose mRNA abundance was not significantly altered by CHIKV infection. (TRANSFAC has no motifs for IRF5). (PDF) [file ppat.1006155.s005.pdf]

S5 Fig.

A

DAY	IRF7	Stat1	IRF3	IRF1	IRF5	IRF8	Stat2	Stat3	IRF9	IRF2	RELA
**2**	83.3	80.2	67.2	61.0	39.6	24.1	19.5	81.8	24.4	30.5	41.1
**7**	71.0	81.1	53.7	60.5	30.0	25.3	19.1	76.5	22.5	29.2	34.6
**30**	55.4	60.4	42.6	31.0	22.7	21.7	14.5	57.2	18.1	15.7	18.3

B

Transcription Factor (TF) name	Day	Transcription Factor ID	Deficit	Total No. search genes	No. search genes with TF site	Total No. background genes	No. bkgrd genes with TF site	Direction	Gene p-value
**IRF7**	2	V$IRF7_01	0.08	816	219	1795	190	Up	1.36E-24
	7	V$IRF7_01	0.1	941	348	2076	483	Up	1.52E-14
	30	V$IRF7_01	0.06	458	57	1175	33	Up	7.44E-13
**STAT1**	2	V$STAT1_Q6	0.11	816	673	1795	1314	Up	1.87E-07
	7	V$STAT1_Q6	0.11	941	781	2076	1571	Up	5.22E-06
	30	V$STAT1_Q6	0.11	458	380	1175	876	Up	2.47E-04
**STAT1:STAT1**	2	V$STAT1STAT1_Q3	0.13	816	281	1795	484	Up	1.17E-04
	7	V$STAT1STAT1_Q3	0.14	941	387	2076	729	Up	0.002
	30	V$STAT1STAT1_Q3	0.14	458	201	1175	403	Up	3.97E-04
**IRF3**	2	V$IRF3_Q3	0.04	816	405	1795	664	Up	1.35E-09
	7	V$IRF3_Q3	0.07	941	741	2076	1463	Up	1.67E-06
	30	V$IRF3_Q3	0.07	458	374	1175	821	Up	8.49E-07
**IRF1**	2	V$IRF1_01	0.06	816	66	1795	49	Up	3.46E-09
	7	V$IRF1_01	0.06	941	64	2076	60	Up	1.45E-06
	30	V$IRF1_01	0.06	458	37	1175	32	Up	6.46E-06
**ICSBP/IRF8**	2	V$ICSBP_Q6	0.11	816	249	1795	237	Up	2.13E-24
	7	V$ICSBP_Q6	0.11	941	248	2076	274	Up	7.70E-18
	30	V$ICSBP_Q6	0.11	458	136	1175	157	Up	1.08E-13
**STAT3**	2	V$STAT3_03	0.11	816	629	1795	1300	Up	0.012
	7	V$STAT3_03	0.12	941	774	2076	1786	Down	0.008
	30	V$STAT3_03	0.11	458	355	1175	844	Up	0.021
**ISGF3**	2	V$ISRE_01	0.08	816	135	1795	79	Up	1.26E-23
	7	V$ISRE_01	0.08	941	126	2076	84	Up	4.21E-19
	30	V$ISRE_01	0.08	458	75	1175	42	Up	3.31E-17
**IRF2**	2	V$IRF2_01	0.12	816	76	1795	102	Up	0.001
	7	V$IRF2_01	0.12	941	79	2076	122	Up	0.012
	30	V$IRF2_01	0.11	458	32	1175	51	Up	0.033
**NF-kappaB**	2	V$NFKB_C	0.06	816	98	1795	85	Up	9.01E-11
	7	V$NFKB_C	0.06	941	95	2076	87	Up	1.49E-09
	30	V$NFKB_C	0.06	458	41	1175	46	Up	1.14E-04
**P50:RELA-P65**	2	V$P50RELAP65_Q5_01	0.07	816	331	1795	581	Up	5.52E-05
	7	V$P50RELAP65_Q5_01	0.05	941	180	2076	256	Up	1.43E-06
	30	V$P50RELAP65_Q5_01	0.03	458	35	1175	38	Up	2.55E-04
