# Supplementary material for: RNA-Seq analysis of chikungunya virus infection and identification of granzyme A as a major promoter of arthritic inflammation
Source: PLoS Pathog. 2017 Feb 16;13(2):e1006155. doi: 10.1371/journal.ppat.1006155 (PMC5312928; doi:10.1371/journal.ppat.1006155)
Supplement: S7 Fig — (A) Formulas for calculating x and y values plotted in Fig 3E. The x axis provides a measure of the proportion of the genes with the putative transcription factor site in their promoter. The y axis provides a measure of the over or under-representation of genes with the putative transcription factor in each IRG gene set. (B) The data plotted in Fig 3E in table form, with transcription factor motif identifiers (TRANFAC) and input/output data provided. (PDF) [file ppat.1006155.s007.pdf]

S7 Fig.

A

	Type II IRG	Background control genes
Number of genes where putative tissue factor site is **not** present	N_s-n_s	N_B-n_B
Number of genes where putative tissue factor site is present	n_s	n_B
Total	N_s	N_B
Proportion	$\frac{n_s+1/2}{N_s+1/2}$	$\frac{n_B+1/2}{N_B+1/2}$

$$\text{x axis} = \frac{1}{2}\log_2\left(\frac{n_S + 1/2}{N_S + 1/2}\right) + \frac{1}{2}\log_2\left(\frac{n_B + 1/2}{N_B + 1/2}\right)$$

$$\text{y axis} = \log_2\left(\frac{n_S + 1/2}{N_S + 1/2} \div \frac{n_B + 1/2}{N_B + 1/2}\right)$$

B

Transcription Factor (TF) ID	Transcription Factor name	Total No. IRGs	No. IRG with TF site	Total No. background genes	No. background genes with TF site	Proportion IRGs with TF site	Proportion background genes with TF site	$-\log_{10}$ gene p-value
**DAY 2**								
V$IRF_Q6	IRF	223	59	1795	35	0.26	0.02	35.2
V$ICSBP_Q6	ICSBP/IRF8	223	102	1795	237	0.46	0.13	26.9
V$IRF7_01	IRF7	223	88	1795	190	0.39	0.11	24.2
V$ISRE_01	ISGF3	223	75	1795	137	0.34	0.08	23.5
V$IRF_Q6_01	IRF	223	76	1795	150	0.34	0.08	22.2
V$STAT1STAT1_Q3	Stat1:Stat1	223	101	1795	595	0.45	0.33	3.4
**DAY 7**								
V$IRF_Q6	IRF	244	54	2076	40	0.22	0.02	30.0
V$ISRE_01	ISGF3	244	37	2076	30	0.15	0.01	19.5
V$IRF_Q6_01	IRF	244	74	2076	175	0.30	0.08	18.8
V$ICSBP_Q6	ICSBP/IRF8	244	93	2076	274	0.38	0.13	18.8
V$IRF7_01	IRF7	244	47	2076	68	0.19	0.03	17.8
V$STAT1STAT1_Q3	Stat1:Stat1	244	105	2076	729	0.43	0.35	1.8
**DAY 30**								
V$IRF_Q6	IRF	155	43	1175	24	0.28	0.02	25.6
V$ISRE_01	ISGF3	155	24	1175	5	0.15	0.00	18.3
V$ICSBP_Q6	ICSBP/IRF8	155	68	1175	157	0.44	0.13	16.8
V$IRF7_01	IRF7	155	35	1175	33	0.23	0.03	16.2
V$IRF_Q6_01	IRF	155	46	1175	106	0.30	0.09	10.7
V$STAT1STAT1_Q3	Stat1:Stat1	155	70	1175	403	0.45	0.34	2.0
