# Supplementary material for: RNA-Seq analysis of chikungunya virus infection and identification of granzyme A as a major promoter of arthritic inflammation
Source: PLoS Pathog. 2017 Feb 16;13(2):e1006155. doi: 10.1371/journal.ppat.1006155 (PMC5312928; doi:10.1371/journal.ppat.1006155)
Supplement: S2 Table — (DOCX) [file ppat.1006155.s011.docx]

**S2 Table.**  Concordance of up-regulated genes identified by RNA-Seq in the current study of CHIKV infected mice and mRNA and protein expression studies in CHIKV infected mice and monkeys.

| **Factor** | **In house RNA-Seq** | | | **Published Literature** | | |
| --- | --- | --- | --- | --- | --- | --- |
|  | **Day 2** | **Day 7** | **Day 30** | **Acute** | **Chronic** | ***In vitro*** |
| ARG1 |  | Ft (▲2.2) |  | Mouse joint/muscle [1,2] |  |  |
| CCL2 (MCP1) | Ft (▲36.6)  LN (▲6.7) | Ft (▲21.6) | Ft (▲3.3) | Mouse sera/joint [3-5]. Monkey plasma [6,7] |  |  |
| CCL4 (MIP1β) | Ft (▲56.6)  LN (▲10.0) | Ft (▲37.4) | Ft (▲4.6) | Mouse sera [5]. Monkey plasma [6] |  |  |
| CCL3 (MIP1α) | Ft (▲16.6)  LN (▲7.0) | Ft (▲11.7)  LN (▲10.7) | Ft (▲3.1) | Mouse joint/sera [4,5]. Monkey plasma [6] |  |  |
| CCL5 (RANTES) | Ft (▲62.8) | Ft (▲41.3) | Ft (▲9.34) | Mouse joint/sera [4,5]. Monkey plasma [6,8] |  |  |
| CCL11 (Eotaxin) | Ft (▲3.8) | Ft (▲2.0) | USR (+1.7) | Mouse sera [5]s |  |  |
| CXCL1 (KC) | Ft (▲5.1) | USR (+2.0) | Ft (▲3.0) | Mouse sera/joint [1,5] |  |  |
| CXCL10 | Ft (▲869.2)  LN (▲16.0) | Ft (▲185.0) | Ft (▲31.4) | Mouse tissue [5] |  |  |
| CXCL11 | Ft (▲116)  LN (▲125) | Ft (▲51.68) | Ft (▲2.08) | Mouse tissue [5] |  |  |
| G-CSF | Ft (▲4.02)  LN (▲6.12) | CP (p=0.00004) | CP (p=0.0013) | Mouse serum and tissue [5] |  |  |
| GM-CSF | Ft (▲11.1) | USR (+7.7) | USR (+5.2) | Mouse sera [5] |  |  |
| IFIT1 | Ft (▲204.6)  LN (▲21.8) | Ft (▲24.8) | Ft (▲7.8) |  |  | MEFs *in vitro* [9] |
| IFNα | Ft (▲2.1x10^6^) | USR (+6.1) | USR (+5.2) | Mouse serum/tissue [3,10]. Monkey plasma [6] |  | Mouse brain cell *in vitro* [11] |
| IFNβ | Ft (▲2.1x10^6^)  LN (▲2.1x10^6^) | USR (+7.7) | USR (+5.3) | Mouse sera [3] |  |  |
| IFNγ | LN (▲10.7) | USR (+6.1) | Ft (▲21.6) | Mouse sera/joint [3] [4,5]. Monkey plasma [6] |  |  |
| IL1β | Ft (▲2.4)  LN (▲2.4) | Ft (▲3.7) | Ft (▲1.4)  USR (+6.3) | Mouse joint/muscle/sera [1,2,5] |  | Mouse brain cell *in vitro* [11] |
| IL1RA | Ft (▲3.5) | Ft (▲2.5) |  | [7] |  |  |
| IL2 | USR (+6.44)  CP (p<0.008) | USR (+7.74)  CP (p=0.00001) | USR (+4.78) | Mouse sera [5]. Monkey plasma [6,7] |  |  |
| IL6 | LN (▲14.4) Ft (▲22.8) | Ft (▲2.2)  USR (+6.3) | Ft (▲2.2)  USR(+4.7) | Mouse sera/muscle/joint [2-5]  Monkey [6,7] |  |  |
| IL9 | USR (+2.3) | USR (+1.70 | USR (+2.0) | Mouse sera [5] |  |  |
| IL10 | Ft (▲16.4) | LN (▲2.2)  Ft (▲30.1) | Ft (▲3.9) | Mouse joint/muscle/sera [1,2,5] |  | Mouse brain cell *in vitro* [11] |
| IL12 | USR (+5.6) | USR (+6.5) | USR (+2.7) |  |  | Mouse brain cell *in vitro* [11] |
| IL15 | Ft (▲5.2) | Ft (▲2.3) | USR (+4.9) | [7] |  |  |
| IL17 | USR (+6.0) | USR (+4.7) | USR (+4.8) | Mouse joint/sera [4,5] |  |  |
| ISG15 | Ft (▲226.8)  LN (▲31.7) | Ft (▲44.6) | Ft (▲4.9) | Mouse skin [12] |  |  |
| ISG54 | Ft (▲75.6)  LN (▲22.1) | Ft (▲10.8) | Ft (▲2.7) | Mouse joint [13] | Mouse joint [13] |  |
| SOCS1 | Ft (▲7.8) | Ft (▲7.8) |  | Mouse muscle [5] |  |  |
| STAT3 | Ft (▲2.88) | Ft (▲1.6)  USR (5.89) | USR (4.80) | Mouse muscle [5] |  |  |
| STAT1 | Ft (▲22.5) | Ft (▲18.6) | Ft (▲2.8) | Mouse muscle [5] |  |  |
| TNFα | Ft (▲4.29) | Ft (▲7.89) | Ft (▲1.8)  USR (+8.4) | Mouse sera/muscle [2,3]. Monkey [6] |  | Mouse brain cell *in vitro* [11] |
| RSAD2 (Viperin) | Ft (▲221.1)  LN (▲28.5) | Ft (▲15.46) | Ft (▲4.42) | Mouse joint [14] |  |  |

Lymph node (LN); Feet (Ft); Fold change (▲); Upstream regulator (USR) with activation z-score; canonical pathway (CP) with p value.

**References**

1. Poo YS, Nakaya H, Gardner J, Larcher T, Schroder WA, Le TT, et al. CCR2 deficiency promotes exacerbated chronic erosive neutrophil-dominated chikungunya virus arthritis. J Virol. 2014;88: 6862-6872.

2. Stoermer KA, Burrack A, Oko L, Montgomery SA, Borst LB, Gill RG, et al. Genetic ablation of arginase 1 in macrophages and neutrophils enhances clearance of an arthritogenic alphavirus. J Immunol. 2012;189: 4047-4059.

3. Gardner J, Anraku I, Le TT, Larcher T, Major L, Roques P, et al. Chikungunya virus arthritis in adult wild-type mice. J Virol. 2010;84: 8021-8032.

4. Dagley A, Ennis J, Turner JD, Rood KA, Van Wettere AJ, Gowen BB, et al. Protection against chikungunya virus induced arthralgia following prophylactic treatment with adenovirus vectored interferon (mDEF201). Antiviral Res. 2014;108: 1-9.

5. Patil DR, Hundekar SL, Arankalle VA. Expression profile of immune response genes during acute myopathy induced by chikungunya virus in a mouse model. Microbes Infect. 2012;14: 457-469.

6. Labadie K, Larcher T, Joubert C, Mannioui A, Delache B, Brochard P, et al. Chikungunya disease in nonhuman primates involves long-term viral persistence in macrophages. J Clin Invest. 2010;120: 894-906.

7. Chen CI, Clark DC, Pesavento P, Lerche NW, Luciw PA, Reisen WK, et al. Comparative pathogenesis of epidemic and enzootic chikungunya viruses in a pregnant Rhesus macaque model. Am J Trop Med Hyg. 2010;83: 1249-1258.

8. Messaoudi I, Vomaske J, Totonchy T, Kreklywich CN, Haberthur K, Springgay L, et al. Chikungunya virus infection results in higher and persistent viral replication in aged rhesus macaques due to defects in anti-viral immunity. PLoS Negl Trop Dis. 2013;7: e2343.

9. Schoggins JW. Interferon-stimulated genes: roles in viral pathogenesis. Curr Opin Virol. 2014;6: 40-46.

10. Schilte C, Couderc T, Chretien F, Sourisseau M, Gangneux N, Guivel-Benhassine F, et al. Type I IFN controls chikungunya virus via its action on nonhematopoietic cells. J Exp Med. 2010;207: 429-442.

11. Das T, Hoarau JJ, Jaffar Bandjee MC, Maquart M, Gasque P. Multifaceted innate immune responses engaged by astrocytes, microglia and resident dendritic cells against chikungunya neuroinfection. J Gen Virol. 2015;96: 294-310.

12. Werneke SW, Schilte C, Rohatgi A, Monte KJ, Michault A, Arenzana-Seisdedos F, et al. ISG15 is critical in the control of chikungunya virus infection independent of UbE1L mediated conjugation. PLoS Pathog. 2011;7: e1002322.

13. Poo YS, Rudd PA, Gardner J, Wilson JA, Larcher T, Colle MA, et al. Multiple immune factors are involved in controlling acute and chronic chikungunya virus infection. PLoS Negl Trop Dis. 2014;8: e3354.

14. Teng TS, Foo SS, Simamarta D, Lum FM, Teo TH, Lulla A, et al. Viperin restricts chikungunya virus replication and pathology. J Clin Invest. 2012;122: 4447-4460.
